# Global genetic prevalence estimates of primary hyperoxaluria are greater than previously reported

**DOI:** 10.1093/ckj/sfaf194

**Published:** 2025-06-18

**Authors:** Giorgia Mandrile, Gill Rumsby, Veronica Sciannameo, Andrea G Cogal, Michelle Glover, John C Lieske, Peter C Harris

**Affiliations:** Genetic Unit and Thalassemia Center, San Luigi Gonzaga University Hospital, Orbassano, Italy; Kintbury United Kingdom, formerly of University College London Hospitals, London, UK; Center for Biostatistics, Epidemiology and Public Health (C-BEPH), Department of Clinical and Biological Sciences, University of Torino, Torino, Italy; Division of Nephrology and Hypertension, Mayo Clinic, Rochester, MN, USA; Novo Nordisk Inc., Plainsboro, NJ, USA; Division of Nephrology and Hypertension, Mayo Clinic, Rochester, MN, USA; Department of Laboratory Medicine and Pathology, Mayo Clinic, Rochester, MN, USA; Division of Nephrology and Hypertension, Mayo Clinic, Rochester, MN, USA

**Keywords:** diagnosis, epidemiology, genetics, primary hyperoxaluria

## Abstract

**Background:**

Primary hyperoxaluria (PH), a rare autosomal recessive disease of oxalate accumulation in the kidneys, is caused by biallelic pathogenic changes in three known genes: *AGXT* (PH1), *GRHPR* (PH2) and *HOGA1* (PH3).

**Methods:**

To better understand the overall risk of developing clinical PH, we manually curated and classified PH genetic variants and calculated the estimated genetic prevalence overall and in five ethnic subpopulations using allelic frequencies from the population Genome Aggregation Database (gnomAD version 2.1.1).

**Results:**

Of the 651 identified PH variants, 273 were found in gnomAD 2.1.1 on the day of download and after reclassification, 208 were determined pathogenic (P) or likely pathogenic (LP) (*AGXT, n* = 94; *GRHPR, n* = 46; and *HOGA1, n* = 68) and a further 65 were classified as rare variants of uncertain significance (VUS). Using P and LP only, estimated carrier frequency was 1:229 for PH1, 1:465 for PH2 and 1:151 for PH3, while genetic prevalence was 1:209 357 for PH1, 1:863 028 for PH2 and 1:90 834 for PH3 (i.e. nearly 5, 1 and 11 per 1 million individuals, respectively). The highest carrier frequencies for *AGXT* pathogenic variants were in East Asians (1 in 146) and the European non-Finnish population (1 in 187); for *GRHPR*, South Asians (1 in 313) and the European non-Finnish population (1 in 413); and for *HOGA1*, Ashkenazi Jewish (1 in 38) and East Asians (1 in 100). The estimated risk of developing PH was ≈1:59 017.

**Conclusions:**

This careful benchmarking exercise indicates that a significant number of individuals at risk for PH symptoms remain undiagnosed. Since these numbers exceed known diagnosed cases of PH, improved screening and diagnosis of this underestimated disease is necessary.

KEY LEARNING POINTS **What was known:**

- Primary hyperoxaluria (PH) is likely underdiagnosed because of broad variability in its age of symptom onset and because the predominant clinical symptom is kidney stones.
- More than 200 variants have been described in the three genes (*AGXT, GRHPR* and *HOGA1*) presumed to cause PH, but for many variants the pathogenicity is unclear or may be incorrectly assigned.

**This study adds:**

- Classification of known globally detected PH genetic variants estimates a risk of developing PH of ≈1:59 017.
- The estimated carrier frequency for the three PH genes is 1:229 (PH1; *AGXT*), 1:465 (PH2; *GRHPR*) and 1:151 (PH3; *HOGA1*).

**Potential impact:**

- The estimated carrier frequencies and disease risk are higher than currently diagnosed in clinical populations, indicating PH remains underdiagnosed.

## INTRODUCTION

The primary hyperoxalurias (PHs) are a family of rare, autosomal recessive metabolic disorders with a shared biochemical phenotype of hepatic oxalate overproduction and abnormally elevated urinary oxalate excretion that often results in recurrent urolithiasis (stones anywhere in the urinary tract, including the kidney [nephrolithiasis]), nephrocalcinosis, progressive chronic kidney disease (CKD) and kidney failure, as well as serious systemic complications of oxalosis [1–11]. Each of the three genetically distinct PH subtypes (PH1, PH2 and PH3) is characterised by a specific enzyme deficiency resulting in increased levels of intrahepatocellular glyoxylate, a direct precursor of oxalate [1, 2, 12–14]. PH1, PH2 and PH3 are caused by mutations in *AGXT* (encoding the liver-specific peroxisomal enzyme alanine-glyoxylate aminotransferase) [12], *GRHPR* (encoding the cytosolic and mitochondrial enzyme glyoxylate reductase/hydroxypyruvate reductase expressed in many body tissues but predominantly active in the liver) [13] and *HOGA1* (encoding liver- and kidney-predominant mitochondrial 4-hydroxy-2-oxoglutarate aldolase), respectively [14].

Current clinical data estimate PH prevalence to be 1–3 per 1 000 000 population, with an incidence rate of 1/120 000 live births [2, 3]. PH is likely underdiagnosed because of broad variability in its age of symptom onset and because the predominant clinical symptom is kidney stones [15]. Signs and symptoms of PH1, the most commonly clinically diagnosed of the three subtypes, range from kidney failure often associated with nephrocalcinosis in early life or later to recurrent calcium oxalate kidney stones throughout the lifespan. PH1 accounts for ≈2% of all kidney failure cases in children [16]. Furthermore, registry data in the USA and Europe reveal that disease manifestations vary by PH type, with kidney failure being very common in PH1 (median age of onset 24–35 years) [17, 18], still common but often occurring later in life in PH2 (median age of onset 40 years) [6] and rarely but still observed in PH3 [9, 10, 19]. However, the known clinical spectrum of all three PH types may change as more patients are identified via increased availability of genetic testing [20–23] and tertiary biochemical analyses [24], as well as the initiation of novel therapies in recent years [11].

More than 200 variants have been described in the *AGXT* gene, with scores of variants described for *GRHPR* and *HOGA1* [4–6, 9, 22, 25]. For many variants the pathogenicity is unclear or may be incorrectly assigned. *In silico* predictions for unique genetic variants are not always accurate, and although relative rarity (typically a minor allele frequency [MAF] of <0.1%) [26] was previously regarded as a moderate level of evidence for pathogenicity, now it is regarded only as supporting evidence [27]. Clearly, accurate determination of PH-causing variants and molecular diagnostics are needed to estimate disease prevalence and thus steer public health policy. A better understanding of the ethnic distribution of PH may also help raise awareness, allowing more informed patient assessment and genetic counselling, formulation of clinical diagnostic guidelines and a better assessment of treatment cost-effectiveness. Most importantly confident assessment of genetic variants is crucial to allow patients to receive life-changing novel therapies that are now available for PH1.

In 2015, an initial attempt to calculate the predicted PH prevalence and carrier frequency was conducted but only included assessed pathogenic variants that had been published or identified from the Rare Kidney Stone Consortium (RKSC) PH registry and was completed at a time when the amount of globally sourced open sequencing data was far less than is available today [5]. This genetic model estimated PH prevalence at 1:58 000 (carrier frequency of 1:70) [5], almost twice the previous estimates suggested by clinical diagnosis [2, 28]. In that analysis, there was a particularly marked difference in expected versus diagnosed cases for PH3, which had a higher carrier frequency than PH1 but was 6-fold less commonly diagnosed in symptomatic patient populations [5]. This observation may have partly reflected the relatively recent identification 5 years earlier of the genetic cause of PH3 [14], as well as underdiagnosis and incomplete penetrance [5]. PH prevalence was also predicted to be approximately equal in European and African American populations since prevalence of the most common European allele, *AGXT* p.Gly170Arg, was roughly balanced by the most common African allele, p.Arg289His [5]. However, p.Arg289His has subsequently been reclassified as a variant of unknown significance (VUS) since it has typically been detected in *cis* with other known pathogenic changes when patients have a clinical phenotype consistent with PH1.

Additional resources are now available to estimate the prevalence of rare monogenic disorders by examining disease-causing variants within populations. The American College of Medical Genetics and Genomics (ACMG) introduced a comprehensive set of guidelines that have standardised criteria for classifying sequence variants in genes associated with Mendelian disorders such as PH [29, 30], and there is an ever-expanding number of new population databases that allow for prevalence calculation across larger ethnic populations. One such information source is the Genome Aggregation Database (gnomAD version 2.1.1), which contains a snapshot in time of the sequence data from >140 000 mainly healthy individuals from case–control studies of adult-onset diseases [26, 31] across five different continental populations (the general population) together with sequence data from 2 populations with distinct disease heritages (i.e. Finnish and Ashkenazi Jewish) [26, 31]. Although PH is a rare recessive disease, making diagnosis and identification of variants difficult, the carrier frequency is significant (see above), so many of the known PH pathogenic variants are likely represented in gnomAD. The frequencies of these variants can be used to determine the minimum genetic prevalence of disease.

Therefore, to estimate genetic prevalence of PH, we curated and systematically classified all known PH genetic variants and determined their allelic frequencies in gnomAD. These prevalence rates were then scaled using ethnic-specific census data from across the world to estimate the population size at risk of PH.

## MATERIALS AND METHODS

### Survey design and oversight

**Figure 1:**
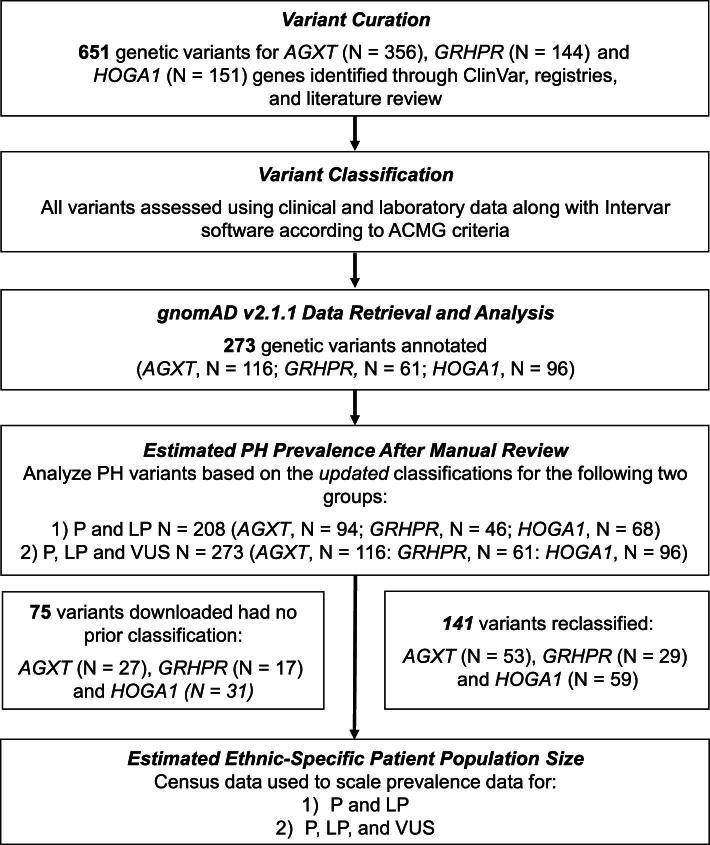
Variant curation and epidemiologic analysis.

This observational study was conducted in three parts: aggregation of PH variants, genetic variant classification and genetic prevalence estimation (Fig. 1). The databases utilized for analyses are regulated by their local institutional review boards, but the present analysis was exempt from institutional review board approval since it utilised only de-identified data.

### Curation of PH genetic variants

Pathogenic variants (P), likely pathogenic variants (LP) and VUS within the three PH loci (*AGXT, GRHPR* and *HOGA1*) were systematically compiled and downloaded from the National Institutes of Health–sponsored ClinVar registry [32, 33] in April 2022. Information on each variant was stored in a predesigned form in Excel (Microsoft, Redmond, WA, USA) prior to manual review.

The OxalEurope [9], RKSC PH [34], gnomAD [26] and TOPMed Bravo [35] databases were searched in July 2022 to supplement identified variants. The OxalEurope [36] and RKSC PH [34] registries were also examined to gather additional phenotypic and functional data that could contribute to substantiating the pathogenicity of any newly identified variants and also those previously catalogued in ClinVar. For completeness, a gnomAD 2.1.1 database search was conducted to identify additional loss-of-function alterations that may not have been present elsewhere.

Finally, an extensive literature review using MEDLINE (via PubMed) and Embase was conducted for PH publications in order to reduce possible bias in frequency calculations and to ensure that all countries worldwide were included. This search was aimed at identifying novel variants not documented in ClinVar. The search algorithms and Boolean strategy included keywords, subject terms and individual gene abbreviations related to PH as follows: ((‘Hyperoxaluria, Primary/genetics’ [MAJR]) OR (‘Primary Hyperoxaluria*’)) AND ((‘Mutation*’) OR (‘genotype’)). The searches were conducted in June 2022 and June 2023 with no limit placed on language or time frame. Eligible articles had abstracts linked to full text reporting original epidemiological research (i.e. cohort and case studies) on PH. All articles deemed relevant after abstract screening underwent full-text screening.

### Genetic variant classification

A systematic classification of genetic variants was executed by employing the rigorous ACMG guidelines and leveraging all available scientific and clinical evidence [29]. This process ensured a robust and standardised assessment of a variant's clinical significance. Not all entries in ClinVar and the literature were initially correctly classified. Thus all variants were reviewed manually, and where additional data were available from registries or the literature, the category was amended accordingly. To reduce investigator bias, missense alterations were evaluated using InterVar, a tool for clinical interpretation of genetic variants based on ACMG guidelines [37], with score adjustment based on additional biochemical and genetic data sourced from diagnostic databases/registry data or published literature. Potential splice site modifications were analysed using the Berkeley Drosophila Genome Project splice Site Prediction by Neural Network software [38]. Furthermore, novel changes identified in the published literature were cross-referenced using Mutalyzer [39] to ensure nomenclature consistency and eliminate the possibility of duplications resulting from non-standard Human Genome Variation Society nomenclature variations [40]. Loss-of-function variants identified from the gnomAD 2.1.1 database encompassed those categorised as ‘stop gained’, ‘frameshift’, ‘splice donor’ and ‘splice acceptor’, with exclusion criteria applied to variants flagged with low confidence [26]. These loss-of-function variants were subsequently classified as P or LP in accordance with ACMG guidelines [29]. Only VUS that were of low allele frequency (MAF <0.001) with clinical information suggestive of PH but insufficient to put the variant in the LP category were included in this study.

### Estimated PH prevalence

To determine the estimated prevalence across geographic regions and diverse ethnic groups, gnomAD 2.1.1 data were downloaded on 25 August 2022 for *AGXT* and 10 November 2022 for *GRHPR* and *HOGA1*. Allelic frequencies were determined after all identified variants were curated and reclassified, which were used to calculate genetic prevalence and risk of developing the disease. Variants in the final classification groups were categorised for assessment as follows: (1) P and LP and (2) P, LP and VUS.

MAF data were obtained from gnomAD [26, 27]. Assuming that pathogenic variants are rare, we divided all gnomAD variants on the basis of their frequency to determine if there were any LP variants not listed in the rare group. The potential unidentified pathogenic variants were evaluated using MAF <0.001.

The Hardy–Weinberg principle was applied to determine the point estimate of the prevalence of disease and carriers [5, 41–43]. The frequency (*p*) of any given variant (*v*) retained as being disease-causing was calculated by dividing the number of alleles bearing the genetic change (*k*) by the total number of alleles subjected to analysis (*n*), i.e. *p = k*/*n.*

The probability of not having the variant (*q*) was computed as *1* − *p* for each variant *v_i_*, i.e. *q_i_* = *1* − *p_i_*. The probability that one or more disease variant will appear was determined by:


\begin{eqnarray*}
E\left[ P \right] = \textit{1} - \prod\limits_{i = 1}^{\left| V \right|} {{q}_i}
\end{eqnarray*}

where *V* is the set of the variants contributing to disease.

The Hardy–Weinberg law (*p*^2^ + 2*pq* + *q*^2^ = 1) states that the estimated disease prevalence is the probability that a disease-causing variant is biallelic [44]:


\begin{eqnarray*}
\textit{Prevalence} = {\left( {\textit{1} - \prod\limits_{i = 1}^{\left| V \right|} {{q}_i} } \right)}^2
\end{eqnarray*}

Counting all biallelic possibilities, carrier frequencies (CFs) were computed as [5]:


\begin{eqnarray*}
CF = \left( {\textit{1} - \textit{prevalence}^2 -{{\left( {\textit{1} - \textit{prevalence}} \right)}}^2} \right).
\end{eqnarray*}

R version 4.2.1 (R Foundation for Statistical Computing, Vienna, Austria) was used for statistical analysis and 90% bias-corrected and accelerated confidence intervals were calculated for each value, with 1000 samples.

### Estimated PH population size

The predominant ethnic group in the African, North American, South American, European, East Asian and South Asian geographic areas was used to estimate the number of individuals at risk for developing PH [31]. The number of patients per million (PPM) was estimated utilizing the prevalence value (1:*n*), where *n* is the population size in each geographic location according to demographic data sourced from the 2022 United Nations Population Prospects report [45]. The PPM formula is defined as PPM = 1 000 000/prevalence (1:*n*). By combining these population projections with prevalence estimates (1:*n*), the comprehensive tally of individuals with a risk of developing PH in each of the six specified geographic regions was derived through the formula: estimated number of individuals = population * (PPM/1 000 000). This methodology ensures a robust estimation of the total number of individuals with a susceptibility to PH, taking into account both prevalence rates and the demographic landscape of each region but not levels of consanguinity.

## RESULTS

### Curation of PH genetic variants

**Figure 2:**
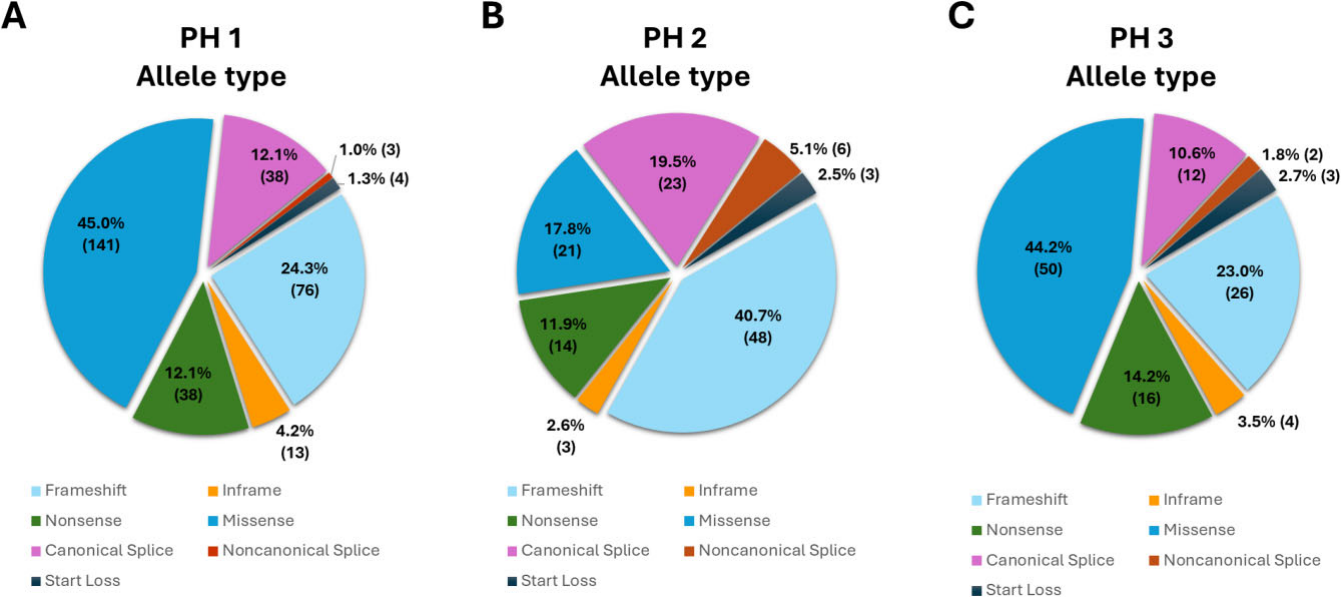
Frequency (% [*n*]) of the different P/LP alleles (*n* = 544) divided by the variant type found in the three PH genes.

To identify possible PH-associated variants, the literature and the ClinVar [32, 33], OxalEurope [9] and RKSC PH [34] registries were screened resulting in a total of 651 variants catalogued: 340 P, 204 LP and 107 rare VUS (Fig. 1). Of the 651 variants, 356 were of *AGXT* (Supplementary Table S1), 144 of *GRHPR* (Supplementary Table S2) and 151 of *HOGA1* (Supplementary Table S3). Following the reclassification processes described in the Methods section, a total of 544 P/LP variants were identified: 313 *AGXT*, 118 *GRHPR* and 113 *HOGA1*. The breakdown of these different pathogenic variant groups in the total PH cohort is shown in Fig. 2.

### Estimated PH prevalence

**Figure 3:**
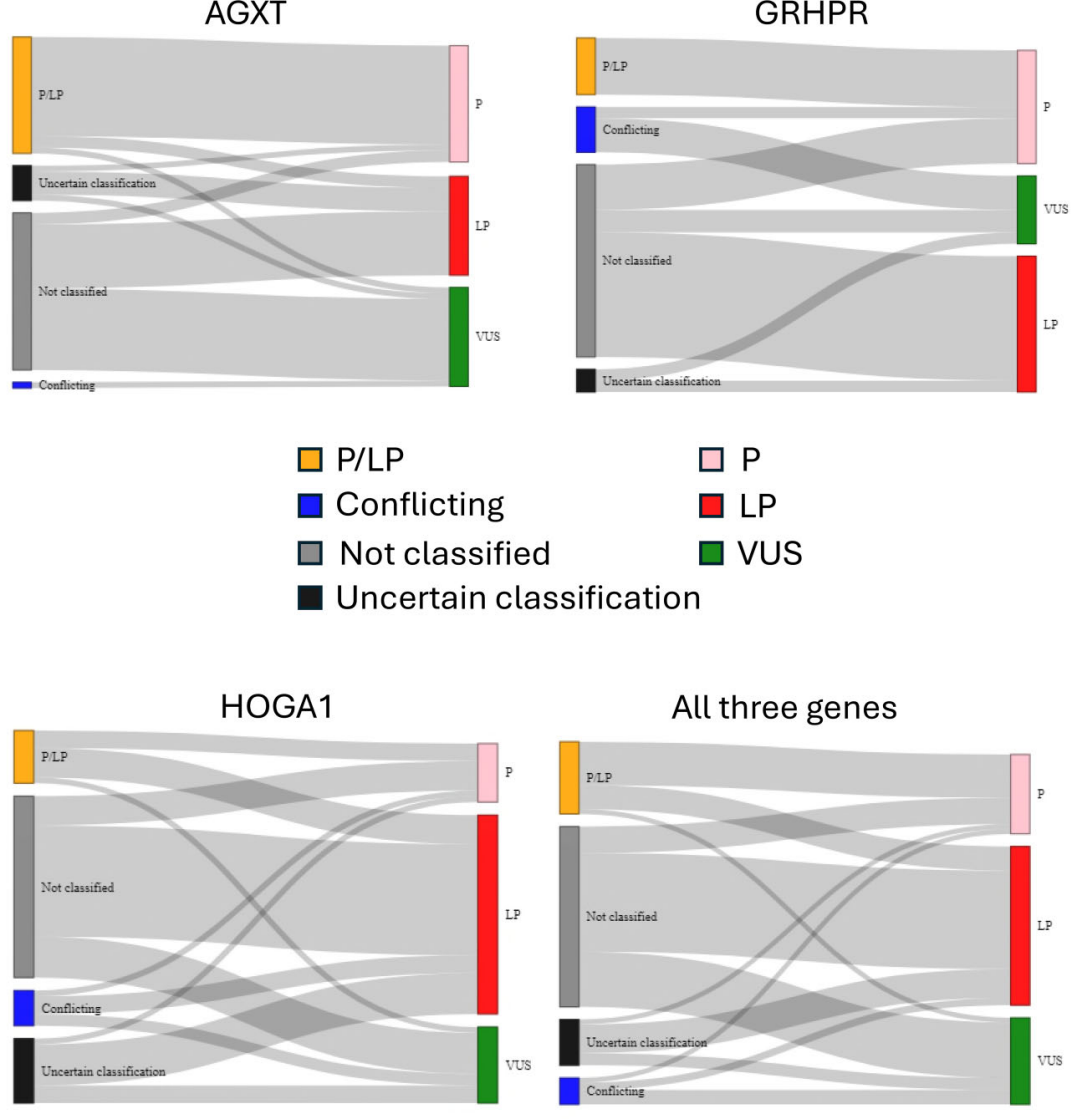
Classification of variants derived from gnomAD version 2.1.1. Sankey plots depict the variants that were reclassified after manual curation.

To estimate the population frequency of significant PH variants, gnomAD 2.1.1 was screened for the 651 PH gene variants and 273 were detected (116 *AGXT*, 61 *GRHPR*, 96 *HOGA1*) (Fig. 1). Among these, 141 variants (53 *AGXT*, 29 *GRHPR*, 59 *HOGA1*) were reclassified (see the Methods section for details; Supplementary Tables S4–S6), including 75 variants (27 *AGXT*, 17 *GRHPR*, 31 *HOGA1*) that had not been previously submitted to ClinVar (Fig. 3). This resulted in a total of 208 P/LP variants (94 *AGXT*, 46 *GRHPR*, 68 *HOGA1*) present in gnomAD 2.1.1.

**Figure 4:**
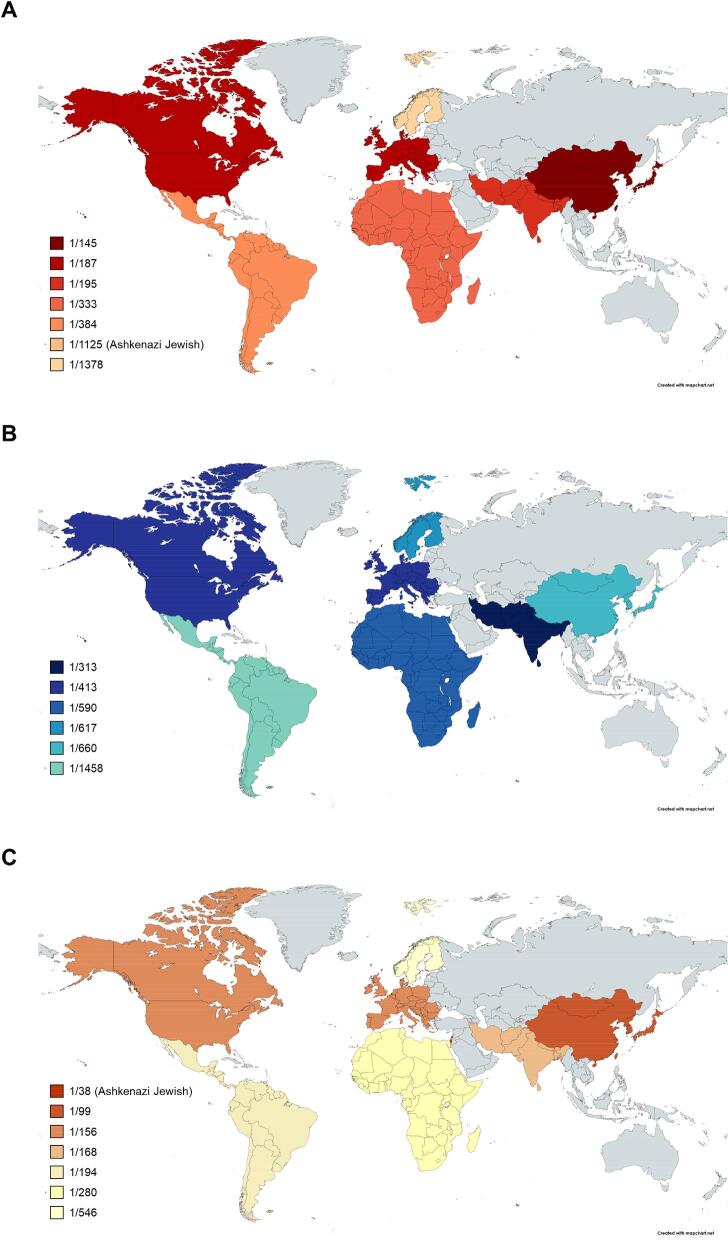
Global and ethnic-specific PH CFs following comprehensive curation and reclassification. **(A)** PH1, **(B)** PH2 and **(C)** PH3 CFs when including pathogenic and likely pathogenic variants only. Deeper heatmap colours indicate greater prevalence.

The P and LP variants detected in gnomAD 2.1.1 were used to calculate the estimated CFs in order to better understand the number of individuals with a high risk of developing PH, resulting in CF rates of 1:229, 1:465 and 1:151 for PH1, PH2 and PH3, respectively (Tables 1–3). The corresponding estimated global genetic prevalence of the PH subtypes was approximately 1:209 357 for PH1, 1:863 028 for PH2 and 1:90 834 for PH3 (i.e. nearly 5, 1 and 11 per 1 million individuals, respectively). PH1, PH2 and PH3 were most prevalent in East Asian (1:84 574), South Asian (1:390 788) and Ashkenazi Jewish (1:5633) populations, respectively (Fig. 4). The global genetic prevalence of PH was ≈1:59 017 regardless of ethnicity, which is ≈17 per 1 million individuals (≈136 000 individuals worldwide) having a high risk of developing PH. Using the estimated prevalence for the predominant ethnic group in each of six geographic regions, regional numbers of individuals at high risk for developing PH were also estimated (Table 4).

**Table 1:** Prevalence and CFs of PH1 mutant alleles found in gnomAD 2.1.1 after manual classification.

Genetic ancestry group	Carriers (90% CI) 1:*n*	Prevalence (90% CI) 1:*n*
P + LP (*n* = 94)		
Overall	229 (140–340)	209 357 (67 442–416 570)
African/African American	334 (202–584)	444 245 (146 413–1 130 384)
Latino American	385 (246–687)	590 388 (223 025–1 523 180)
Ashkenazi Jewish	1226 (483–5035)	6 006 663 (791 033–101 364 624)
European Finnish	1379 (582–12 230)	7 596 223 (885 457–32 005 055)
European non-Finnish	187 (91–342)	139 473 (26 110–375 133)
East Asian	146 (73–331)	84 574 (16 707–264 193)
South Asian	195 (129–320)	151 843 (59 573–338 116)
Other	188 (119–344)	141 065 (47 451–356 061)
P + LP + VUS (*n* = 116)		
Overall	129 (78–210)	65 862 (21 491–145 246)
African/African American	69 (33–220)	18 927 (3251–98 720)
Latino American	161 (80–372)	102 488 (17 469–318 405)
Ashkenazi Jewish	67 (24–613)	17 876 (1031–111 881)
European Finnish	617 (311–1840)	1 520 938 (292 034–5 493 766)
European non-Finnish	136 (79–236)	73 921 (21 328–172 763)
East Asian	128 (71–261)	64 573 (16 004–183 957)
South Asian	184 (125–293)	134 847 (54 915–293 467)
Other	94 (51–190)	34 669 (8237–102 648)

CI: confidence interval.

**Table 2:** Prevalence and CFs of PH2 mutant alleles found in gnomAD 2.1.1 after manual classification.

Genetic ancestry group	Carriers (90% CI) 1:*n*	Prevalence (90% CI) 1:*n*
P + LP (*n* = 46)		
Overall	465 (305–712)	863 028 (319 574–1 745 278)
African/African American	591 (353–1247)	1 394 151 (434 671–3 966 391)
Latino American	1459 (878–3475)	8 505 623 (2 766 835–25 079 836)
Ashkenazi Jewish	Undefined	Undefined
European Finnish	618 (223–10 824)	1 522 892 (70 296–1 941 068)
European non-Finnish	413 (206–905)	681 174 (133 447–2 141 496)
East Asian	662 (256–3326)	1 749 627 (180 876–14 884 397)
South Asian	313 (149–957)	390 788 (48 005–1 772 939)
Other	863 (386–3606)	2 973 869 (517 664–37 650 496)
P + LP + VUS (*n* = 61)		
Overall	228 (136–391)	207 465 (65 602–510 305)
African/African American	103 (40–772)	41 929 (3554–317 389)
Latino American	360 (166–975)	515 567 (80 963–2 123 225)
Ashkenazi Jewish	109 (55-undefined)	46 674 (2958–undefined)
European Finnish	618 (172–5412)	1 522 892 (77 866–18 584 894)
European non-Finnish	272 (164–520)	295 271 (90 044–782 306)
East Asian	321 (156–1196)	410 684 (67 887–2 002 663)
South Asian	222 (110–511)	195 893 (38 255–609 579)
Other	349 (1.80–1024)	486 317 (114 870–1 939 527)

CI: confidence interval; undefined: not possible to calculate using the available data.

**Table 3:** Prevalence and CFs of PH3 mutant alleles found in gnomAD 2.1.1 after manual classification.

Genetic ancestry group	Carriers (90% CI) 1:*n*	Prevalence (90% CI) 1:*n*
P + LP (*n* = 68)		
Overall	151 (79–270)	90 834 (21 426–213 858)
African/African American	281 (185–443)	314 119 (122 941–652 868)
Latino American	194 (95–473)	149 811 (23 645–532 832)
Ashkenazi Jewish	38 (16–315)	5633 (593–51 977)
European Finnish	546 (217–6208)	1 191 725 (145 046–7 762 989)
European non-Finnish	156 (68–463)	96 734 (9543–127 866)
East Asian	100 (59–211)	39 501 (10 911–111 254)
South Asian	168 (93–317)	112 401 (28 906–317 402)
Other	114 (60–259)	51 899 (11 225–157 220)
P + LP + VUS (*n* = 96)		
Overall	127 (76–209)	64 113 (19 632–150 897)
African/African American	232 (160–353)	213 989 (92 954–444 113)
Latino American	157 (85–323)	98 569 (22 482–316 783)
Ashkenazi Jewish	37 (15–179)	5471 (569–39 131)
European Finnish	525 (221–6208)	1 094 326 (136 693–7 762 989)
European non-Finnish	133 (65–323)	70 454 (10 257–255 934)
East Asian	71 (47–130)	19 702 (7346–51 824)
South Asian	146 (89–281)	84 422 (26 337–220 658)
Other	93 (55–172)	33 880 (10 166–90 976)

CI: confidence interval.

**Table 4:** Estimated number of individuals at high risk for developing PH in specific geographic regions [45].

Location^a^	Population (as of 1 July 2022)	PH1 calculated prevalence (1:*n*)	PH1 PPM	Estimated number of individuals with a high risk of developing PH1	PH2 calculated prevalence (1:*n*)	PH2 PPM	Estimated number of individuals with a high risk of developing PH2	PH3 calculated prevalence (1:*n*)	PH3 PPM	Number of individuals with a high risk of developing PH3
Africa	1 393 676 000	444 245	2.3	3137	1 394 151	0.7	1000	314 119	3.2	4437
East Asia	1 663 696 000	84 574	11.8	19 671	1 749 627	0.6	951	39 501	25.3	42 118
South Asia	1 989 452 000	151 843	6.6	13 102	390 787	2.6	5091	112 401	8.9	17 700
North America	375 278 001	139 473	7.2	2691	681 174	1.5	551	96 734	10.3	3879
South America	434 254 000	590 388	1.7	736	8 505 623	0.1	51	149 811	6.7	2899
Europe	745 173 000	139 473	7.2	5343	681 174	1.5	1094	96 734	10.3	7703

a Designated subpopulations that align with ethnic group from gnomAD 2.1.1 [26].

Rare VUS were added to the analysis, resulting in 259 total variants (109 *AGXT*, 56 *GRHPR*, 94 *HOGA1*) in gnomAD 2.1.1, to better understand the number of individuals potentially at risk for developing PH. The resulting estimated CFs of PH1, PH2 and PH3 were 1:129, 1:228 and 1:127, respectively, with a global genetic prevalence of 1:65 862 for PH1, 1:207 465 for PH2 and 1:64 113 for PH3 (Tables 1–3). The estimated global genetic prevalence of PH was ≈1:28 089 regardless of ethnicity, which translates to 36 per 1 million individuals (≈286 000 individuals worldwide) at risk of developing PH in their lifetime (Supplementary Fig. S1). Ashkenazi Jewish and African/African American individuals had the highest CFs for PH1 pathogenic variants/VUS (1 in 67 and 1 in 69, respectively) and PH2 pathogenic variants/VUS (1 in 109 and 1 in 103, respectively), and Ashkenazi Jewish and East Asians had the highest CFs for PH3 pathogenic variants/VUS (1 in 37 and 1 in 71, respectively).

## DISCUSSION

Despite numerous articles describing PH genetics and clinical manifestations, robust epidemiology data have not been available until now. A prior analysis of PH prevalence completed nearly 10 years ago was based on the limited number of published PH pathogenic variants and those identified in the RKSC database at that time, which were then annotated in the relatively small National Heart, Lung, and Blood Institute Exome Sequencing Project database for prevalence calculations [5]. The current updated analysis builds upon that prior one by curating and ascertaining the classification of genetic variants from the largest, fully genotyped PH cohort to date and using a far larger population database. Thus our findings represent the most robust estimations of worldwide PH epidemiology to date.

The current study included curation and classification of epidemiologic genetic variant sequence data sourced from ClinVar and the RKSC and OxalEurope registries, along with other general population databases. Conducting this extensive exercise required an international collaborative effort made possible by the diligence and cooperation of many investigators who have shared and reported their data. Our results now represent a benchmark for ongoing epidemiologic research into PH, which will lead to a better understanding of disease prevalence.

The extensive manual classification effort identified new PH pathogenic variants, confirmed the importance of ongoing variant evaluation, underlined the expected frequency of PH by ethnic group and geography and suggests that PH3 is the most genetically common form of PH. There were no individual variants that disproportionately contributed to the increased prevalence of any PH type compared with the ClinVar classification analysis and corresponding CF data reported by Hopp *et al.* [5] (PH1: 1 in 128, 153 and 195, respectively; PH2: 1 in 228, 444 and 279, respectively; PH3: 1 in 127, 138 and 185, respectively). Rather, the increase in predicted prevalence occurred because variants previously misclassified in ClinVar and noted in the gnomAD databases have been reclassified appropriately together with newly captured and classified variants added to the overall increase in PH estimates. All updated variant information has been submitted to the ClinVar registry so clinicians and scientists will have access to robust information about each variant and its potential pathogenicity. With continued identification of new disease-causing genetic variants, enhanced understanding of VUS variants and continued updates to the various population databases such as gnomAD, there will be an ongoing need to update and re-evaluate this work.

The high predicted number of carriers in our study emphasizes the importance of considering genetic testing in patients with relevant clinical symptoms suggesting PH and/or recurrent stone formation. Ethnicity is important since East Asians are at the highest risk of PH1, South Asians for PH2 and Ashkenazi Jewish individuals for PH3. These findings have profound implications regarding routine PH genetic screening and, particularly in the large East Asian and South Asian populations, are important in raising awareness to allow for early identification and appropriate PH management before renal dysfunction occurs. Further research is needed to clarify data on penetrance of disease in subpopulations with pathogenic variants in the *HOGA1* gene, given available data in the Ashkenazi Jewish population [46]. In addition, PH risk in the Middle Eastern population is unclear, but the newest version of gnomAD (4.0.0) includes individuals within this ethnic group. A better understanding of the impact of ethnicity is likely with the continued improvement of these large population databases.

Although the current estimate of overall PH prevalence after manual variant classification was similar to the prior Hopp *et al.* [5] analysis (1:59 017 versus 1:58 000, respectively), the Hopp *et al.* analysis included the p.Arg289His variant, which is no longer considered pathogenic. Note that removal of this variant decreased PH1 prevalence by 48% (CF 1 in 270, prevalence 1:291 256) and overall PH prevalence by 18% (CF 1 in 79, prevalence 1:71 333) in the Hopp study [5].

It should be emphasized that only around half of the newly curated variants were previously found in ClinVar, suggesting that the prevalence of these disorders is even higher and more extensive than previously suspected. Thus population data are needed to correctly estimate the disease prevalence. Prevalence may also be impacted by other considerations, such as the need for a variant to be on a specific haplotype to result in pathogenicity, as for some genetic variants and the minor/major haplotype on *AGXT*, and the lack of data regarding whole exons/gene changes (copy number variants) in gnomAD. However, deletional variants are rare and would have a very small impact on the estimates, similar to base pair changes not listed in gnomAD. Hopefully, analysis with third-generation sequencers will allow re-evaluation of disease prevalence considering the *AGXT* haplotype. Since PH is an autosomal recessive disease, the inbred coefficient of a single population could change the disease prevalence, especially in countries with a high frequency of consanguineous marriages or in geographic isolation. The same can be said for immigrant populations who often recreate their communities in the foreign country. Because of this phenomenon, even if marriages are not between close relatives, the resulting prevalence of recessive disorders can still be higher than predicted based on gene frequency alone, as has been observed in the UK with a heightened PH2 frequency in the Pakistani immigrant community [6]. The discrepancy in prevalence data extracted from ClinVar and diagnosis-based prevalence is more pronounced for PH3, possibly due to an underdiagnosis of milder phenotypes and a lack of disease penetrance. Severe PH cases (i.e. infantile PH) may also lead to premature death before a diagnosis is reached, and individuals affected by severe paediatric-onset disease, as well as their first-degree relatives, are excluded from ClinVar [31].

In this analysis, the risk for prevalence overestimation was mitigated by excluding benign and likely benign variants. Weak VUS (downloaded by ClinVar without other information) were also not included in the analysis. All known variants in the three PH genes were evaluated not only on the basis of *in silico* prediction, but also in light of clinical and *in vitro* data where and when such data were available. The calculations were run with and without stronger VUS (i.e. variants that did not quite meet the scoring threshold for LP), because some VUS may contribute to disease and eventually be identified as pathogenic. Indeed, many variants in our study were moved from VUS to P/LP through our research, highlighting that while VUS variants may not initially be known to be disease-causing, they also should not be discounted. Enrolling a patient with clinical symptoms of PH with a VUS genetic report in a registry study is invaluable in helping clinicians and researchers better understand how that variant contributes to a patient's phenotype. Ultimately this clarification of the VUS variant not only helps the patient but can profoundly impact a large population of individuals who may carry the same variant.

In conclusion, these findings foster a better understanding of PH genetic prevalence and suggest that a significant number of individuals living with PH remain undiagnosed and not adequately treated. Improving the screening and diagnosis of this underestimated disease with postnatal screening and screening in adults with relevant symptoms is essential for tailoring treatment for specific types of PH.

## Supplementary Material

sfaf194_Supplemental_File

## Data Availability

All data supporting the findings of this study are provided within the publication and its supplementary material.

## References

[1] Hoppe B. An update on primary hyperoxaluria. Nat Rev Nephrol 2012;8:467–75. 10.1038/nrneph.2012.113 22688746

[2] Cochat P, Rumsby G. Primary hyperoxaluria. N Engl J Med 2013;369:649–58. 10.1056/NEJMra1301564 23944302

[3] Sas DJ, Harris PC, Milliner DS. Recent advances in the identification and management of inherited hyperoxalurias. Urolithiasis 2019;47:79–89. 10.1007/s00240-018-1093-3 30535828

[4] Harambat J, Fargue S, Acquaviva C et al. Genotype-phenotype correlation in primary hyperoxaluria type 1: the p.Gly170Arg AGXT mutation is associated with a better outcome. Kidney Int 2010;77:443–9. 10.1038/ki.2009.435 20016466

[5] Hopp K, Cogal AG, Bergstralh EJ et al. Phenotype-genotype correlations and estimated carrier frequencies of primary hyperoxaluria. J Am Soc Nephrol 2015;26:2559–70. 10.1681/ASN.2014070698 25644115 PMC4587693

[6] Garrelfs SF, Rumsby G, Peters-Sengers H et al. Patients with primary hyperoxaluria type 2 have significant morbidity and require careful follow-up. Kidney Int 2019;96:1389–99. 10.1016/j.kint.2019.08.018 31685312

[7] Monico CG, Rossetti S, Belostotsky R et al. Primary hyperoxaluria type III gene HOGA1 (formerly DHDPSL) as a possible risk factor for idiopathic calcium oxalate urolithiasis. Clin J Am Soc Nephrol 2011;6:2289–95. 10.2215/CJN.02760311 21896830 PMC3358997

[8] Allard L, Cochat P, Leclerc AL et al. Renal function can be impaired in children with primary hyperoxaluria type 3. Pediatr Nephrol 2015;30:1807–13. 10.1007/s00467-015-3090-x 25972204

[9] Martin-Higueras C, Garrelfs SF, Groothoff JW et al. A report from the European Hyperoxaluria Consortium (OxalEurope) Registry on a large cohort of patients with primary hyperoxaluria type 3. Kidney Int 2021;100:621–35. 10.1016/j.kint.2021.03.031 33865885

[10] Singh P, Viehman JK, Mehta RA et al. Clinical characterization of primary hyperoxaluria type 3 in comparison with types 1 and 2. Nephrol Dial Transplant 2022;37:869–75. 10.1093/ndt/gfab027 33543760 PMC9214566

[11] Groothoff JW, Metry E, Deesker L et al. Clinical practice recommendations for primary hyperoxaluria: an expert consensus statement from ERKNet and OxalEurope. Nat Rev Nephrol 2023;19:194–211. 10.1038/s41581-022-00661-1 36604599

[12] Danpure CJ, Jennings PR, Watts RW. Enzymological diagnosis of primary hyperoxaluria type 1 by measurement of hepatic alanine: glyoxylate aminotransferase activity. Lancet 1987;329:289–91. 10.1016/S0140-6736(87)92023-X 2880111

[13] Cramer SD, Ferree PM, Lin K et al. The gene encoding hydroxypyruvate reductase (GRHPR) is mutated in patients with primary hyperoxaluria type II. Hum Mol Genet 1999;8:2063–9. 10.1093/hmg/8.11.2063 10484776

[14] Belostotsky R, Seboun E, Idelson GH et al. Mutations in DHDPSL are responsible for primary hyperoxaluria type III. Am Hum Genet 2010;87:392–9. 10.1016/j.ajhg.2010.07.023 PMC2933339 20797690

[15] Milliner DS, Harris PC, Sas DJ et al. Primary hyperoxaluria type 1. In: Adam MP, Feldman J, Mirzaa GM et al., eds. GeneReviews. Seattle: University of Washington, 1993–2025. 26401545

[16] Hulton SA. The primary hyperoxalurias: a practical approach to diagnosis and treatment. Int J Surg 2016;36:649–54. 10.1016/j.ijsu.2016.10.039 27815184

[17] Bergstralh EJ, Monico CG, Lieske JC et al. Transplantation outcomes in primary hyperoxaluria. Am J Transplant 2010;10:2493–501. 10.1111/j.1600-6143.2010.03271.x 20849551 PMC2965313

[18] Metry EL, Garrelfs SF, Deesker LJ et al. Determinants of kidney failure in primary hyperoxaluria type 1: findings of the European Hyperoxaluria Consortium. Kidney Int Rep 2023;8:2029–42. 10.1016/j.ekir.2023.07.025 37849991 PMC10577369

[19] Richard E, Blouin JM, Harambat J et al. Late diagnosis of primary hyperoxaluria type III. Ann Clin Biochem 2017;54:406–11. 10.1177/0004563216677101 27742850

[20] Gefen AM, Sethna CB, Cil O et al. Genetic testing in children with nephrolithiasis and nephrocalcinosis. Pediatr Nephrol 2023;38:2615–22. 10.1007/s00467-023-05879-0 36688940 PMC11071637

[21] Knoers N, Antignac C, Bergmann C et al. Genetic testing in the diagnosis of chronic kidney disease: recommendations for clinical practice. Nephrol Dial Transplant 2022;37:239–54. 10.1093/ndt/gfab218 34264297 PMC8788237

[22] Mandrile G, Beck B, Acquaviva C et al. Genetic assessment in primary hyperoxaluria: why it matters. Pediatr Nephrol 2023;38:625–34. 10.1007/s00467-022-05613-2 35695965 PMC9842587

[23] Breeggemann MC, Harris PC, Lieske JC et al. How we treat primary hyperoxaluria type 1. Clin J Am Soc Nephrol 2024;19:800–2. 10.2215/CJN.0000000000000460 38494457 PMC11168812

[24] Woodward G, Pryke R, Hoppe B et al. Rapid liquid chromatography tandem mass-spectrometry screening method for urinary metabolites of primary hyperoxaluria. Ann Clin Biochem 2019;56:232–9. 10.1177/0004563218811365 30373392

[25] Rumsby G, Williams E, Coulter-Mackie M. Evaluation of mutation screening as a first line test for the diagnosis of the primary hyperoxalurias. Kidney Int 2004;66:959–63. 10.1111/j.1523-1755.2004.00842.x 15327387

[26] Francioli L, Tiao G, Karczewski K et al. gnomAD v2.1. https://gnomad.broadinstitute.org/news/2018-10-gnomad-v2-1/ (13 August 2024, date last accessed).

[27] Karczewski KJ, Francioli LC, Tiao G et al. The mutational constraint spectrum quantified from variation in 141,456 humans. Nature 2020;581:434–43. 10.1038/s41586-020-2308-7 32461654 PMC7334197

[28] van Woerden CS, Groothoff JW, Wanders RJ et al. Primary hyperoxaluria type 1 in The Netherlands: prevalence and outcome. Nephrol Dial Transplant 2003;18:273–9. 10.1093/ndt/18.2.273 12543880

[29] Richards S, Aziz N, Bale S et al. Standards and guidelines for the interpretation of sequence variants: a joint consensus recommendation of the American College of Medical Genetics and Genomics and the Association for Molecular Pathology. Genet Med 2015;17:405–24. 10.1038/gim.2015.30 25741868 PMC4544753

[30] ClinGen. Sequence variant interpretation. https://clinicalgenome.org/working-groups/sequence-variant-interpretation/ (13 August 2024, date last accessed).

[31] Gudmundsson S, Singer-Berk M, Watts NA et al. Variant interpretation using population databases: lessons from gnomAD. Hum Mutat 2022;43:1012–30. 10.1002/humu.24309 34859531 PMC9160216

[32] National Library of Medicine. ClinVar. https://www.ncbi.nlm.nih.gov/clinvar/ (13 August 2024, date last accessed).

[33] Landrum MJ, Lee JM, Benson M et al. ClinVar: improving access to variant interpretations and supporting evidence. Nucleic Acids Res 2018;46:D1062–7. 10.1093/nar/gkx1153 29165669 PMC5753237

[34] Rare Kidney Stone Consortium. Primary hyperoxaluria. https://www.rarekidneystones.org/hyperoxaluria/ (13 August 2024, date last accessed).

[35] TOPMed. Bravo. https://bravo.sph.umich.edu/freeze8/hg38/ (13 August 2024, date last accessed).

[36] European Consortium of Hyperoxaluria. Home page. https://oxal-europe.org/ (13 August 2024, date last accessed).

[37] Li Q, Wang K. InterVar: clinical interpretation of genetic variants by the 2015 ACMG-AMP guidelines. Am Hum Genet 2017;100:267–80. 10.1016/j.ajhg.2017.01.004 PMC5294755 28132688

[38] Berkeley Drosophila Genome Project. Splice site prediction by neural network. https://fruitfly.org/seq_tools/splice.html (13 August 2024, date last accessed).

[39] LUMC Mutalyzer 3. Mutalyzer tool suite. https://mutalyzer.nl/ (13 August 2024, date last accessed).

[40] Human Genome Variation Society. About the society. https://www.hgvs.org/ (13 August 2024, date last accessed).

[41] Borges P, Pasqualim G, Giugliani R et al. Estimated prevalence of mucopolysaccharidoses from population-based exomes and genomes. Orphanet J Rare Dis 2020;15:324. 10.1186/s13023-020-01608-0 33208168 PMC7672855

[42] Hughes BG, Harrison PM, Hekimi S. Estimating the occurrence of primary ubiquinone deficiency by analysis of large-scale sequencing data. Sci Rep 2017;7:17744. 10.1038/s41598-017-17564-y 29255295 PMC5735152

[43] Park KS. Carrier frequency and predicted genetic prevalence of Pompe disease based on a general population database. Mol Genet Metab Rep 2021;27:100734. 33717985 10.1016/j.ymgmr.2021.100734 PMC7933537

[44] Clark WT, Yu GK, Aoyagi-Scharber M et al. Utilizing ExAC to assess the hidden contribution of variants of unknown significance to Sanfilippo type B incidence. PLoS One 2018;13:e0200008. 10.1371/journal.pone.0200008 29979746 PMC6034809

[45] United Nations Department of Economic and Social Affairs. Population Division. World population prospects 2022. https://population.un.org/wpp/ (13 August 2024, date last accessed).

[46] Bar R, Ben-Shalom E, Duvdevani M et al. Mutations in *HOGA1* do not confer a dominant phenotype manifesting as kidney stone disease. J Urol 2021;205:1394–9. 10.1097/JU.0000000000001528 33350326

