# Supplementary material for: Global genetic prevalence estimates of primary hyperoxaluria are greater than previously reported
Source: Clin Kidney J. 2025 Jun 18;18(7):sfaf194. doi: 10.1093/ckj/sfaf194 (PMC12378434; doi:10.1093/ckj/sfaf194)
Supplement: sfaf194_Supplemental_File [file sfaf194_Supplemental_File.docx]

**Supplementary Appendix**

This appendix has been provided by the authors to give readers additional information about their work.

Supplement to: Giorgia Mandrile, Gill Rumsby, Veronica Sciannameo et al. Global Genetic Prevalence Estimates of Primary Hyperoxaluria are Greater Than Previously Reported

## **SUPPLEMENTARY RESULTS**

**Table S1:** *AGXT* variants (P, LP, VUS) identified and analyzed, and their corresponding effect.^a^

| Variant | Effect | Class |
| --- | --- | --- |
| c.1_524del | p.M1fs | P |
| c.1_358del | p.Met1fs | P |
| c.2_3delinsAT | p.Met1Asn | P |
| c.2T>C | p.Met1? | P |
| c.3G>T | p.Met1? | P |
| c.3G>A | p.Met1? | LP |
| c.22G>C | p.Val8Leu | P |
| c.23_24delinsAT | p.Val8Asp | LP |
| c.26_27insA | p.Lys12fs | P |
| c.28C>T | p.Pro10Ser | P |
| c.28C>G | p.Pro10Ala | VUS |
| c.28_29delCCinsA | p.Pro10Thrfs*36 | P |
| c.31_32insT | p.Pro11fs | LP |
| c.32C>G | p.Pro11Arg | P |
| c.33del | p.Lys12fs | P |
| c.32_33del | p.Pro11Glnfs*156 | P |
| c.33dup | p.Lys12fs | P |
| c.33_34dup | p.Ala13Argfs*34 | P |
| c.35A>G | p.Lys12Arg | VUS |
| c.40dup | p.Leu14Profs*154 | P |
| c.52C>T | p.Leu18Phe | VUS |
| c.67C>T | p.Gln23* | P |
| c.70C>A | p.Leu24Ile | VUS |
| c.74T>G | p.Leu25Arg | P |
| c.77T>C | p.Leu26Pro | P |
| c.79G>T | p.Gly27Trp | LP |
| c.80G>A | p.Gly27Glu | VUS |
| Variant | Effect | Class |
| c.82C>T | p.Pro28Ser | VUS |
| c.83del | p.Pro28fs | P |
| c.106C>T | p.Arg36Cys | P |
| c.107G>A | p.Arg36His | P |
| c.116_117dup | p.Ala40fs | P |
| c.121G>A | p.Gly41Arg | P |
| c.121G>T | p.Gly41Trp | P |
| c.121dup | p.Gly41Argfs*127 | P |
| c.122G>A | p.Gly41Glu | P |
| c.122G>T | p.Gly41Val | P |
| c.125G>A | p.Gly42Glu | P |
| c.126del | p.Leu43fs | P |
| c.126dup | p.Leu43fs | P |
| c.130C>T | p.Gln44* | P |
| c.139G>A | p.Gly47Arg | P |
| c.139G>C | p.Gly47Arg | LP |
| c.139G>T | p.Gly47Trp | LP |
| c.140G>A | p.Gly47Glu | LP |
| c.166-11_166-8delinsTGCATGCAAGAT | p.Ile56fs | LP |
| c.166-1G>A | p.? | P |
| c.166-1_172dup | p.? | P |
| c.167T>A | p.Ile56Asn | P |
| c.172G>A | p.Asp58Asn | VUS |
| c.174C>A | p.Asp58Glu | VUS |
| c.175G>A | p.Glu59Lys | P |
| c.175G>T | p.Glu59* | P |
| c.187G>C | p.Gly63Arg | P |
| Variant | Effect | Class |
| c.188G>A | p.Gly63Asp | LP |
| c.190A>T | p.Ile64Phe | LP |
| c.191T>A | p.Ile64Asn | LP |
| c.198C>A | p.Tyr66* | P |
| c.203T>C | p.Phe68Ser | VUS |
| c.205C>T | p.Gln69* | P |
| c.209C>A | p.Thr70Asn | P |
| c.214A>C | p.Asn72His | VUS |
| c.214A>G | p.Asn72Asp | LP |
| c.215dup | p.Asn72fs | P |
| c.215A>T | p.Asn72Ile | LP |
| c.221_227dup | p.Val77fs | P |
| c.224C>A | p.Thr75Lys | VUS |
| c.224delC | p.Thr75Asnfs*43 | P |
| c.238_239insTTGCCAA | p.Gly80Valfs*90 | P |
| c.242C>A | p.Ser81* | P |
| c.242C>G | p.Ser81Trp | LP |
| c.242C>T | p.Ser81Leu | P |
| c.244G>C | p.Gly82Arg | P |
| c.244G>A | p.Gly82Arg | LP |
| c.245G>A | p.Gly82Glu | P |
| c.245_249delinsAGA | p.Gly82Glufs*85 | P |
| c.248A>G | p.His83Arg | P |
| c.254C>A | p.Ala85Asp | P |
| c.260_271del | p.Glu87_Leu90del | P |
| c.265G>A | p.Ala89Thr | VUS |
| c.276del | p.Asn92fs | P |
| Variant | Effect | Class |
| c.278_279insCACACTT | p.Leu94Thrfs*76 | P |
| c.283_285dup | p.Glu95dup | P |
| c.283G>A | p.Glu95Lys | P |
| c.285G>T | p.Glu95Asp | VUS |
| c.286C>G | p.Pro96Ala | VUS |
| c.292G>C | p.Asp98His | LP |
| c.299_307dup | p.Val102_Gly103ins  ValLeuVal | LP |
| c.302T>C | p.Leu101Pro | P |
| c.304G>T | p.Val102Phe | VUS |
| c.305T>A | p.Val102Asp | LP |
| c.307G>A | p.Gly103Arg | LP |
| c.322T>C | p.Trp108Arg | P |
| c.323G>A | p.Trp108* | P |
| c.324G>T | p.Trp108Cys | P |
| c.326G>T | p.Gly109Val | P |
| c.326G>A | p.Gly109Glu | LP |
| c.327del | p.Gln110fs | P |
| c.331C>T | p.Arg111* | P |
| c.332G>A | p.Arg111Gln | P |
| c.335C>A | p.Ala112Asp | P |
| c.341A>G | p.Asp114Gly | VUS |
| c.346G>T | p.Gly116Arg | LP |
| c.346G>A | p.Gly116Arg | P |
| c.349G>T | p.Glu117* | P |
| c.349dup | p.Glu117Glyfs*51 | P |
| c.352C>A | p.Arg118Ser | LP |
| c.352C>T | p.Arg118Cys | LP |
| c.353G>A | p.Arg118His | P |
| c.355A>G | p.Ile119Val | VUS |
| c.358G>A | p.Gly120Arg | LP |
| Variant | Effect | Class |
| c.358+1G>T | p.? | P |
| c.358+2T>G | p.? | P |
| c.359-1_382del | p.? | P |
| c.359-2A>G | p.? | LP |
| c.364C>T | p.Arg122* | P |
| c.371A>C | p.His124Pro | P |
| c.374_381del | p.Pro125fs | P |
| c.389del | p.Pro130Leufs*24 | LP |
| c.402C>G | p.Tyr134* | P |
| c.406_410dup | p.Gln137fs | P |
| c.409C>T | p.Gln137* | P |
| c.416_418del | p.Val139del | P |
| c.423+1G>A | p.? | LP |
| c.423+2T>C | p.? | LP |
| c.423G>C | p.Glu141Asp | LP |
| c.423G>T | p.Glu141Asp | P |
| c.424-2A>G | p.? | P |
| c.445del | p.Val149fs | P |
| c.447_454del | p.Leu151fs | P |
| c.449T>C | p.Leu150Pro | P |
| c.454T>A | p.Phe152Ile | P |
| c.455T>G | p.Phe152Cys | VUS |
| c.455T>C | p.Phe152Ser | VUS |
| c.457T>G | p.Leu153Val | P |
| c.458T>A | p.Leu153* | P |
| c.460del | p.Thr154fs | P |
| c.466G>A | p.Gly156Arg | P |
| c.466G>C | p.Gly156Arg | P |
| c.467G>C | p.Gly156Ala | VUS |
| c.469G>C | p.Glu157Gln | VUS |
| Variant | Effect | Class |
| c.469G>T | p.Glu157* | P |
| c.469del | p.Glu157fs | P |
| c.473C>A | p.Ser158* | P |
| c.473C>T | p.Ser158Leu | LP |
| c.481G>A | p.Gly161Ser | P |
| c.481G>C | p.Gly161Arg | P |
| c.481G>T | p.Gly161Cys | P |
| c.482G>A | p.Gly161Asp | P |
| c.484G>A | p.Val162Met | VUS |
| c.491A>G | p.Gln164Arg | VUS |
| c.497T>C | p.Leu166Pro | P |
| c.506T>C | p.Phe169Ser | LP |
| c.507_508insA | p.Gly170fs | P |
| c.508G>A | p.Gly170Arg | P |
| c.517T>C | p.Cys173Arg | LP |
| c.518G>A | p.Cys173Tyr | P |
| c.519_520delinsGA | p.Cys173_His174  delinsTrpAsn | P |
| c.519C>A | p.Cys173T* | P |
| c.524+1G>A | p.? | LP |
| c.524+2T>A | p.? | LP |
| c.525-2A>G | p.? | LP |
| c.525-2A>T | p.? | P |
| c.525-1G>A | p.? | P |
| c.533G>A | p.Cys178Tyr | P |
| c.534C>G | p.Cys178Trp | LP |
| c.536T>C | p.Leu179Pro | VUS |
| c.547G>A | p.Asp183Asn | P |
| c.551C>A | p.Ser184* | P |
| c.551C>T | p.Ser184Leu | VUS |
| c.557_562delinsATCGGT | p.Ala186_Ser187  delinsAspArg | P |
| Variant | Effect | Class |
| c.560_561insGGT | p.Ser187_Leu188insVal | P |
| c.560C>A | p.Ser187Tyr | LP |
| c.560C>T | p.Ser187Phe | P |
| c.568G>A | p.Gly190Arg | P |
| c.569G>T | p.Gly190Leu | LP |
| c.569G>C | p.Gly190Ala | LP |
| c.569_570insA | p.Thr191Aspfs* | LP |
| c.570del | p.Thr191fs | P |
| c.577del | p.Leu193fs | P |
| c.577dup | p.Leu193fs | P |
| c.583A>C | p.Met195Leu | P |
| c.584T>G | p.Met195Arg | P |
| c.585G>A | p.Met195Ile | LP |
| c.593A>C | p.Gln198Pro | VUS |
| c.595G>A | p.Gly199Ser | P |
| c.595+1G>A | p.? | LP |
| c.595+1G>T | p.? | LP |
| c.595+2T>G | p.? | P |
| c.596-2A>G | p.? | P |
| c.597_601del | p.Ile200Hisfs*23 | LP |
| c.598A>G | p.Ile200Val | P |
| c.601G>A | p.Asp201Asn | LP |
| c.602A>T | p.Asp201Val | LP |
| c.603C>A | p.Asp201Glu | P |
| c.605T>A | p.Ile202Asn | P |
| c.612C>A | p.Tyr204* | P |
| c.613T>C | p.Ser205Pro | P |
| c.614C>A | p.Ser205Ter | P |
| c.614C>T | p.Ser205Leu | P |
| c.622C>T | p.Gln208* | P |
| Variant | Effect | Class |
| c.627G>T | p.Lys209Asn | LP |
| c.628G>C | p.Ala210Pro | P |
| c.632T>G | p.Leu211Arg | LP |
| c.637G>A | p.Ala213Thr | VUS |
| c.638C>A | p.Ala213Asp | VUS |
| c.642_645del | p.Pro215fs | P |
| c.642del | p.Pro215fs | P |
| c.646G>A | p.Gly216Arg | P |
| c.653C>T | p.Ser218Leu | LP |
| c.653C>A | p.Ser218* | LP |
| c.658A>T | p.Ile220Phe | P |
| c.661T>C | p.Ser221Pro | P |
| c.662_664del | p.Ser221del | LP |
| c.667A>C | p.Ser223Arg | LP |
| c.673_676del | p.Lys225fs | LP |
| c.676G>A | p.Ala226Thr | VUS |
| c.679_680+2del | p.? | P |
| c.680+1G>T | p.? | P |
| c.680+1G>A | p.? | P |
| c.680+1G>C | p.? | P |
| c.680+2T>A | p.? | P |
| c.680+480_776+69  delinsTGAGA | p.? | P |
| c.680+5G>C | p.? | P |
| c.681-1G>A | p.? | LP |
| c.681-1G>T | p.? | LP |
| c.682_683del | p.Lys228Glufs*26 | P |
| c.683_685AGA[1] | p.Lys229del | LP |
| c.683_685AGA[3] | p.Lys229dup | LP |
| c.686_688del | p.Lys229del | LP |
| c.693C>G | p.Tyr231* | P |
| Variant | Effect | Class |
| c.697C>T | p.Arg233Cys | P |
| c.698G>A | p.Arg233His | P |
| c.698G>T | p.Arg233Leu | P |
| c.716_718del | p.Ser239del | P |
| c.725dup | p.Asp243fs | P |
| c.727G>C | p.Asp243His | P |
| c.731T>C | p.Ile244Thr | P |
| c.731T>A | p.Ile244Asn | P |
| c.733_734del | p.Lys245Valfs*9 | P |
| c.737G>A | p.Trp246* | P |
| c.738G>A | p.Trp246* | P |
| c.743C>T | p.Ala248Val | LP |
| c.744del | p.Asn249fs | P |
| c.751_752delinsAA | p.Trp251Lys | P |
| c.751T>C | p.Trp251Arg | LP |
| c.752G>A | p.Trp251* | P |
| c.752delinsAA | p.Trp251* | P |
| c.753G>A | p.Trp251* | P |
| c.757T>C | p.Cys253Arg | P |
| c.766delC | p.Gln256fs16* | P |
| c.774G>T | p.Arg258Ser | VUS |
| c.776+1G>A | p.? | P |
| c.776+1G>C | p.? | P |
| c.776+2T>G | p.? | LP |
| c.777-1G>C | p.? | P |
| c.777-2A>G | p.? | P |
| c.779A>G | p.Tyr260Cys | LP |
| c.781C>G | p.His261Asp | LP |
| c.783T>A | p.His261Gln | P |
| c.795del | p.Val266fs | P |
| Variant | Effect | Class |
| c.798_802delins  ACAATCTCAG | p.Ile267fs | P |
| c.799A>T | p.Ile267Phe | VUS |
| c.806T>C | p.Leu269Pro | P |
| c.807dup | p.Tyr270Valfs*62 | P |
| c.815T>C | p.Leu272Pro) | VUS |
| c.822G>C | p.Glu274Asp | P |
| c.823_824dup | p.Ser275Argfs*38 | P |
| c.823_824del | p.Ser275fs | P |
| c.823A>C | p.Ser275Arg | P |
| c.824G>A | p.Ser275Asn | LP |
| c.824G>T | p.Ser275Ile | LP |
| c.826_827delinsGA | p.Leu276Glu | LP |
| c.827T>A | p.Leu276Gln | VUS |
| c.829_830delinsA | p.Ala277Aspfs*55 | P |
| c.829_830insA | p.Ala277fs | P |
| c.830delinsAA | p.AlaGlufs*55 | P |
| c.832del | p.Leu278Sfs | P |
| c.834del | p.Ile279fs | P |
| c.[32C>T:836T>C] | p.Ile279Thr | P |
| c.838del | p.Ala280fs | LP |
| c.841G>T | p.Glu281* | P |
| c.844C>T | p.Gln282* | P |
| c.845A>G | p.Gln282Arg | LP |
| c.846+?_c.1179+?del | p.Gly283fs | P |
| c.846+1G>A | p.? | P |
| c.847-12_-6del | p.Gly283fs | LP |
| c.847-1G>C | p.? | P |
| c.847-2_847-1del | p.? | LP |
| c.847-3C>G | p.G283? | P |
| c.851T>C | p.Leu284Pro | P |
| Variant | Effect | Class |
| c.853G>T | p.Glu285* | P |
| c.858C>G | p.Asn286Lys | VUS |
| c.860_861delinsCG | p.Ser287Thr | P |
| c.864G>A | p.Trp288* | P |
| c.866G>A | p.Arg289His | VUS |
| c.869_882dup | p.Ala294Serfs21* | P |
| c.872A>C | p.His291Pro | LP |
| c.886_888del | p.Ala296del | P |
| c.891T>G | p.Tyr297* | P |
| c.893T>C | p.Leu298Pro | P |
| c.901_903delinsGCG | p.Arg301Ala | VUS |
| c.907C>T | p.Gln303* | P |
| c.910delG | p.Ala304Hisfs*8 | P |
| c.917G>T | p.Gly306Val | VUS |
| c.919del | p.Leu307fs | P |
| c.922C>T | p.Gln308* | P |
| c.941C>T | p.Pro314Leu | VUS |
| c.942+1G>A | p.Pro314? | P |
| c.942+1G>T | p.? | P |
| c.942G>A | p.Pro314=;?splice | VUS |
| c.943-1G>A | p.? | P |
| c.943-1G>T | p.? | P |
| c.947T>C | p.Leu316Pro | P |
| c.949C>T | p.Arg317Trp | LP |
| c.956C>T | p.Pro319Leu | P |
| c.959_960del | p.Thr320Serfs | P |
| c.961G>T | p.Val321Phe | VUS |
| c.971_972del | p.Val324fs | P |
| c.973del | p.Ala325fs* | P |
| c.976del | p.Val326fs | P |
| Variant | Effect | Class |
| c.983_988del | p.Ala328_Tyr330  delinsAsp | P |
| c.992A>T | p.Asp331Val | VUS |
| c.994_995del | p.Trp332Glufs*14 | P |
| c.995G>A | p.Trp332* | P |
| c.996G>A | p.Trp332* | P |
| c.997A>T | p.Arg333* | P |
| c.1007T>A | p.Val336Asp | P |
| c.1014C>G | p.Tyr338* | P |
| c.1015delG | p.Val339Serfs | P |
| c.1016T>C | p.Val339Ala | VUS |
| c.1045G>A | p.Gly349Ser | P |
| c.1048G>A | p.Gly350Ser | VUS |
| c.1049G>A | p.Gly350Asp | P |
| c.1071+1G>A | p.? | P |
| c.1072_1079del | p.Val358fs | LP |
| c.1076T>C | p.Leu359Pro | P |
| c.1072–2A>G | p.? | LP |
| c.1078C>T | p.Arg360Trp | LP |
| c.1079G>A | p.Arg360Gln | P |
| c.1079G>C | p.Arg360Pro | LP |
| c.1084G>A | p.Gly362Ser | LP |
| c.1087_1095del | p.Leu363_Gly365del | VUS |
| c.1093G>T | p.Gly365Cys | P |
| c.1093_1094del | p.Gly365Leufs*47 | LP |
| c.1094G>A | p.Gly365Asp | VUS |
| c.1094G>C | p.Gly365Ala | LP |
| c.1102G>A | p.Ala368Thr | P |
| c.1110_1111delCG[1] | p.Asn372fs | P |
| c.1120G>C | p.Asp374His | LP |
| c.1124G>A | p.Arg375His | VUS |
| Variant | Effect | Class |
| c.1125_1126del | p.Val376fs | P |
| c.1148C>A | p.Ala383Asp | P |
| c.1151T>C | p.Leu384Pro | P |
| c.1157del | p.His386Profs*143 | LP |
| c.1161C>A | p.Cys387* | P |
|  |  |  |
|  |  |  |
|  |  |  |
|  |  |  |
|  |  |  |
|  |  |  |
|  |  |  |
|  |  |  |
|  |  |  |
|  |  |  |
|  |  |  |

^a^Nucleotide numbering based on NM_000030.3 (*AGXT* cDNA) and NG_008005.1 (genomic).

gnomAD: Genome Aggregation Database; LP: likely pathogenic variant; P: pathogenic variant; VUS: variant of unknown significance.

**Table S2:** *GRHPR* variants (P, LP, VUS) identified and analyzed, and their corresponding effect.^a^

| Variant | Effect | Class |
| --- | --- | --- |
| c.[-4_-3delinsAT] | p.M1ext-4 | P |
| c.1A>G | p.Met1? | P |
| c.1A>T | p.M1? | LP |
| c.2T>G | p.M1? | LP |
| c.13C>T | p.Arg5* | P |
| c.34A>G | p.Thr12Ala | VUS |
| c.43A>C | p.Ile15Leu | VUS |
| c.45del | p.Ala17fs | P |
| c.68T>C | p.Leu23Pro | P |
| c.83+1G>C | p? | LP |
| c.[84-13_84-5delinsCTTT] | p.? | P |
| c.[84-8_84-5del;84-13_84-12del] | p.? | P |
| c.84-8_84-5del | p.? | VUS |
| c.84-2A>G | p.? | P |
| c.92_103del | p.Val31_Trp34del | VUS |
| c.102G>A | p.Trp34Ter | P |
| c.103del | p.D35Tfs*11 | P |
| c.107C>A | p.Ser36Ter | P |
| c.109_118delinsCAC | p.Ala37fs7* | P |
| c.119T>C | p.Ile40Thr | VUS |
| c.136G>T | p.Glu46Ter | P |
| c.139C>T | p.Arg47Ter | P |
| c.154del | p.Ala52Profs*56 | P |
| c.181G>A | p.Asp61Asn | LP |
| c.188_189del | p.Val63Glyfs*29 | LP |
| c.203T>C | p.Leu68Pro | P |
| c.211G>A | p.Ala71Thr | VUS |
| c.215_493del | p.Ala73_Gly65del | P |
| Variant | Effect | Class |
| c.214+1G>T | p.? | LP |
| c.214+2T>G | p.? | LP |
| c.215-13_215-7del | p.? | VUS |
| c.215-2_215-1del | p.? | LP |
| c.228dup | Val77Serfs*16 | LP |
| c.248_249del | p.Val83Glyfs*9 | P |
| c.258_272del | p.His87_Asp91del | LP |
| c.271del | p.Asp91Metfs*17) | P |
| c.274G>T | p.Glu92Ter | P |
| c.277del | p.Ile93Serfs*15 | P |
| c.286C>T | p.Arg96Cys | VUS |
| c.286_287+1del | p.? | VUS |
| c.287_287+4delinsCCC | p.Arg96fs | P |
| c.287+2T>C | p.? | LP |
| c.287G>A | p.Arg96His | LP |
| c.287G>T | p.Arg96Leu | LP |
| c.288-1G>C | p.? | LP |
| c.288-2_288del | p.? | P |
| c.295C>T | p.Arg99Ter | P |
| c.306C>A | p.Tyr102Ter | LP |
| c.337G>A | p.Glu113Lys | LP |
| c.337G>T | p.Glu113Ter | P |
| c.344C>A | p.Ala115Glu | LP |
| g.[8888_8892delins  7465_7744;8897_8910dup] | p.Val116? | P |
| c.346_347dup | p.Leu118Profs*17 | P |
| c.348del | p.Ser117Profs | LP |
| g.8888_8890delins7465_7748 | p.? | P |
| Variant | Effect | Class |
| c.351_373del | p.Leu118Valfs*65 | LP |
| c.356_369dup | p.Arg124Tyrfs*15 | P |
| c.361dup | p.Thr121Asnfs*70 | P |
| c.370C>T | p.Arg124Gly | LP |
| c.375del | p.Leu126Cysfs*8 | P |
| c.403_404+1del | p.? | VUS |
| c.404+3_404+6del | c.404+3_404+6del | P |
| c.404+5G>A | p.? | LP |
| c.404del | p.Asn135Metfs*30 | LP |
| c.405-1G>A | p.? | LP |
| c.412T>C | p.Trp138Arg | VUS |
| c.435_436del | p.Trp145Cysfs*45 | LP |
| c.441_442del | p.Cys147Trpfs*43 | LP |
| c.454dup | p.Thr152Asnfs*39 | P |
| c.456_464del | p.Gln153_Thr155del | VUS |
| c.457C>T | p.Gln153Ter | LP |
| c.478G>A | p.Gly160Arg | P |
| c.493+2T>A | p.? | P |
| c.494G>A | p.Gly165Asp | P |
| c.494-2A>G | p.? | P |
| c.496del | p.Gln166Argfs*7 | LP |
| c.501del | p.Ile168Leufs*5 | P |
| c.511C>T | p.Arg171Cys | VUS |
| c.515del | p.Leu172Argfs*47 | LP |
| c.532_533del | p.Gln178Glufs*12 | P |
| c.532dup | p.Gln178Profs*13 | P |
| c.536_537del | p.Ser179Thrfs*9 | LP |
| c.540del | p.Leu181Cysfs*38 | P |
| c.589del | p.Ala197Glnfs*22 | P |
| Variant | Effect | Class |
| c.593_594del | p.Glu198Valfs*6 | P |
| c.597del | p.Phe199Leufs*20 | LP |
| c.598+2delT | p.? | LP |
| c.598+1del | p.? | LP |
| c.598+1G>T | p.? | LP |
| c.598+2dup | p.? | LP |
| c.599-1G>C | p.? | LP |
| c.608_609del | p.Pro203Argfs*7 | P |
| c.617_671del | p.Ala206Aspfs*11 | P |
| c.626C>T | p.Ser209Phe | LP |
| c.694del | p.Gln232Argfs*3 | P |
| c.701T>C | p.Met234Thr | VUS |
| c.734+1G>A | p.? | LP |
| c.735-1G>A | p.? | P |
| c.735-2A>G | p.? | LP |
| c.735-2delA | p.? | LP |
| c.742G>A | p.Val248Ile | VUS |
| c.743T>A | p.Val248Asp | LP |
| c.748A>T | p.Asn250Tyr | VUS |
| c.755dup | p.Asp252Glufs*11 | LP |
|  |  |  |
| Variant | Effect | Class |
| c.759C>G | p.Asp253Glu | VUS |
| c.761T>C | p.Leu254Pro | LP |
| c.766C>T | p.Gln256Ter | P |
| c.769dup | p.Ala257Glyfs*6 | P |
| c.780dup | p.Gly261Trpfs*2 | LP |
| c.781_782delinsTAC | p.Gly261Tyrfs*2 | P |
| c.783del | p.Lys262Argfs*9 | P |
| c.783dup | p.Lys262Ter | LP |
| c.788T>C | p.Ile263Thr | VUS |
| c.806A>G | p.Asp269Gly | LP |
| c.824del | p.Pro275Hisfs*9 | P |
| c.847del | p.Leu283Ter | P |
| c.849dup | p.Thr284Aspfs*28 | LP |
| c.863del | p.Cys288Leufs*2 | LP |
| c.865+2dup | p.? | LP |
| c.865+1G>T | p.? | LP |
| c.866_867del | p.Val289Aspfs*22 | P |
| c.866-2A>G | p.? | LP |
| c.866-34_866-8 | p? | LP |
|  |  |  |
|  |  |  |
| Variant | Effect | Class |
| c.867_870dup | p.Leu291Aspfs*22 | LP |
| c.872T>C | p.Leu291Pro | VUS |
| c.874C>T | p.Pro292Ser | VUS |
| c.889G>A | p.Ala297Thr | LP |
| c.890_891dup | p.Thr298Profs*21 | P |
| c.904C>T | p.Arg302Cys | P |
| c.905G>A | p.Arg302His | P |
| c.905G>C | p.Arg302Pro | LP |
| c.930_931dup | p.Asn311Lysfs*8 | P |
| c.932dup | p.Asn311Lysfs*14 | LP |
| c.934A>G | p.Asn312Asp | P |
| c.935dup | p.Asn312Lysfs*13 | VUS |
| c.954_955del | p.Glu320Alafs*4 | VUS |
| c.954del | [p.Glu320Serfs*47](https://www.ncbi.nlm.nih.gov/snp/rs1057516831) | LP |
| c.955G>T | p.Gly319Trp | VUS |
| c.958G>T | p.Glu320Ter | LP |
| c.961C>G | p.Pro321Ala | VUS |
| c.964_965del | p.Met322Alafs*2 | VUS |
| c.965T>C | p.Met322Thr | P |
| c.965T>G | p.Met322Arg | P |
| c.986A>G | p.Ter329Trp | VUS |

^a^Nucleotide numbering based on NM_012203.1 (*GRHPR* cDNA) and NG_008135.1 (genomic).

gnomAD: Genome Aggregation Database; LP: likely pathogenic variant; P: pathogenic variant; VUS: variant of unknown significance.

**Table S3:** *HOGA1* variants (P, LP, VUS) identified and analyzed, and their corresponding effect.^a^

| Variant | Effect | Class |
| --- | --- | --- |
| c.2T>G | p.Met1? | P |
| c.3G>A | p.Met1? | P |
| c.3G>T | p.Met1? | P |
| c.10_11insTGGTC | p.Pro4Leufs*9 | P |
| c.25_27del | p.Ser9del | VUS |
| c.33G>T | p.Arg11Ser | VUS |
| c.41T>A | p.Leu14Gln | VUS |
| c.64G>A | p.Val22Met | VUS |
| c.70delG | p.Val24Serfs*19 | P |
| c.75G>A | Trp25* | LP |
| c.85G>T | p.Glu29* | P |
| c.94_96del | p.Lys32del | VUS |
| c.103A>G | p.Ile35Val | VUS |
| c.106G>A | p.Ala36Thr | VUS |
| c.107C>T | p.Ala36Val | LP |
| c.110G>A | p.Gly37Asp | P |
| c.110G>T | p.Gly37Val | LP |
| c.117C>A | pTyr.39* | P |
| c.119C>A | p.Pro40His | VUS |
| c.122del | p.Pro41Leufs*2 | LP |
| c.122_123del | p.Pro41Argfs*47 | LP |
| c.122_135del | Pro41fs | P |
| c.122dup | p.Val42Cysfs*r47 | LP |
| c.123del | p.Val42* | P |
| c.133C>G | p.Pro45Ala | VUS |
| c.134C>G | p.Pro45Arg | LP |
| c.134C>T | p. Pro45Leu | P |
| Variant | Effect | Class |
| c.139A>C | p.Thr47Pro | VUS |
| c.158delA | p.Asp53Alafs*32 | P |
| c.185_186del | p.His62Glnfs*26 | P |
| c.189delA | p.Lys63Asnfs*22 | P |
| c.206T>G | p.Phe69Cys | LP |
| c.208C>T | p.Arg70* | P |
| c.209G>C | p.Arg70Pro | P |
| c.212-1G>A | p.? | LP |
| c.212G>T | p.Gly71Val | LP |
| c.221T>G | p.Val74Gly | LP |
| c.227G>A | p.Gly76Asp | LP |
| c.234T>A | p.Asn78Lys | LP |
| c.238G>T | p.Glu80* | P |
| c.238G>A | p.Glu80Lys | VUS |
| c.250del | p.Leu84* | LP |
| c.251T>C | p.Leu84Pro | P |
| c.260G>C | p.Ser87Thr | VUS |
| c.266G>A | p.Arg89His | LP |
| c.289C>T | p.Arg97Cys | LP |
| c.290G>T | p.Arg97Leu | VUS |
| c.290G>A | p.Arg97His | P |
| c.308A>T | p.Asn103Ile | P |
| c.317T>A | p.Leu106Gln | VUS |
| c.331G>A | p.Gly111Arg | LP |
| c.336C>G | p.Cys112Trp | VUS |
| c.337G>A | p.Glu113Lys | P |
| c.340+1G>A | p.? | LP |
| c.340+2T>C | p.? | LP |
| Variant | Effect | Class |
| c.341-1G>A | p.? | LP |
| c.344C>G | p.Thr115Ser | VUS |
| c.346C>T | p.Gln116* | P |
| c.356T>A | p.Val119Glu | LP |
| c.356T>G | p.Val119Gly | LP |
| c.376G>A | p.Ala126Thr | VUS |
| c.385G>A | p.Gly129Arg | LP |
| c.388delG | p.Ala130LeufsTer7 | P |
| c.397del | p.Ala133fs | P |
| c.398C>T | p.Ala133Val | VUS |
| c.404del | p.Val135fs | P |
| c.407T>C | p.Val136Ala | VUS |
| c.413del | p.Pro138LeufsTer8 | LP |
| c.415T>G | p.Cys139Gly | VUS |
| c.420C>G | p.Tyr140* | P |
| c.425G>A | p.Arg142His | LP |
| c.443C>T | p.Ala148Val | VUS |
| c.448del | p.Leu150SerfsTer46 | LP |
| c.469-1G>C | p.? | LP |
| c.487A>T | p.Ile163Phe | VUS |
| c.520A>G | p.Thr174Ala | VUS |
| c.529G>T | p.Asp177Tyr | LP |
| c.533T>A | p.Leu178Gln | LP |
| c.533T>C | p.Leu178Pro | P |
| c.533_535delinsCGA | p.Leu178_Pro179delinsProThr | LP |
| c.535C>A | p.Pro179Thr | LP |
| c.554C>T | p.Thr185Met | VUS |
| Variant | Effect | Class |
| c.568C>G | p.Pro190Ala | LP |
| c.569C>T | p.Pro190Leu | P |
| c.573del | p.Asn191fs | P |
| c.580G>A | p.Gly194Ser | LP |
| c.604-1G>C | p.? | LP |
| c.634A>G | p.Thr212Ala | LP |
| c.634A>C | p.Thr212Pro | LP |
| c.635C>A | p.Thr212Asn | VUS |
| c.651dup | p.Gln218Serfs*57 | LP |
| c.653del | p.Gln218fs | P |
| c.660G>C | p.Leu220Phe | P |
| c.661G>C | p.Ala221Pro | VUS |
| c.661G>A | p.Ala221Thr | VUS |
| c.700G>T | p.Gly234* | P |
| c.700+2T>A | p.? | LP |
| c.700+2T>G | p.? | LP |
| c.700+5G>T | p.? | P |
| c.701-2A>G | p.? | LP |
| c.713del | p.Gly238Alafs*42 | LP |
| c.713dup | p.Val239fs | P |
| c.720C>A | p.Cys240* | LP |
| c.728C>A | p.Ala243Asp | P |
| c.733G>A | p.Val25Ile | P |
| c.733G>T | p.Val245Phe | LP |
| c.743del | p.Ala248Valfs*32 | LP |
| c.745C>G | p.Gln249Glu | VUS |
| c.754C>T | p.Gln252* | P |
| c.763C>T | pArg255* | P |
| c.769_770del | p.Cys257Leufs*17 | P |
|  |  |  |
| Variant | Effect | Class |
| c.769T>G | p.Cys257Gly | LP |
| c.770G>A | p.Cys257Tyr | LP |
| c.776C>T | p.Thr259Met | VUS |
| c.785G>A | p.Trp262* | LP |
| c.787G>T | p.Gln263* | LP |
| c.793G>A | p.Ala265Thr | VUS |
| c.796C>T | p.Gln266* | P |
| c.803_805del | p.Leu268del | P |
| c.811C>T | p.Arg271Cys | VUS |
| c.812G>A | p.Arg271His | LP |
| c.814C>G | p.Leu272Val | LP |
| c.818T>C | p.Ile273Thr | LP |
| c.823C>A | p.Pro275Thr | VUS |
| c.832G>A | p.Ala278Thr | VUS |
| c.833C>A | p.Ala278Glu | VUS |
| c.834_834+1delinsTT | p.? | P |
| c.834+1G>A | p.? | LP |
| c.834+1G>T | p.? | P |
| c.834G>A | p.Ala278= (? splicing) | P |
| c.839C>T | p.Thr280Ile | P |
| c.841C>T | p.Arg281Trp | LP |
| c.844C>T | p.Arg282Cys | LP |
| c.845G>A | p.Arg282His | VUS |
| c.860G>A | p.Gly287Glu | P |
| c.860G>T | p.Gly287Val | P |
| c.872T>C | p.Ile291Thr | VUS |
| c.875T>C | p.Met292Thr | P |
| c.881G>A | p.Trp294* | P |
| c.899del | p.Gly300Alafs*10 | LP |
|  |  |  |
| Variant | Effect | Class |
| c.904del | p.Cys302Alafs*8 | P |
| c.907C>T | p.Arg303Cys | P |
| c.908G>A | p.Arg303His | LP |
| c.926T>C | p.Leu309Pro | LP |
| c.940G>T | p.Glu314* | P |
| c.943_954dup | p.Glu315_Arg318dup | LP |
| c.944_946del | p.Glu315del | P |
| c.949_956dup | p.Met319Ilefs*22; | LP |
| c.953G>A | p.Arg318His | VUS |
| c.960G>T | p.Gly287Val | LP |
| c.973G>A | p.Gly325Ser | P |
| c.977G>A | p.Trp326* | LP |
|  |  |  |

^a^Nucleotide numbering based on NM_138413.3 (*HOGA1*).

gnomAD: Genome Aggregation Database; LP: likely pathogenic variant; P: pathogenic variant; VUS: variant of unknown significance.

**Table S4:** Classification of *AGXT* variants yielded from gnomAD v.2.1.1 before and after manual review.

| **Variant** | **Protein consequence** | **ClinVar classification** | **After manual classification** | **ACMG criteria** | | | **Allele frequency** | **Reference** |
| --- | --- | --- | --- | --- | --- | --- | --- | --- |
| c.2T>C | p.Met1Thr | P/LP | P | PVS1 PM2_supporting PM3PP3 PP4 | | | 4.57E-05 | ^S1^ ^S2^  ^S1,2^ |
| c.32C>G | p.Pro11Arg | LP | P | PS3 PM2_supporting PM3_strong PP3 PP4 | | | 0.000155 | ^S2-4^  ^S2-4^ |
| c.106C>T | p.Arg36Cys | P/LP | P | PS3 PM1 PM2_supporting PP3 PP4 | | | 4.04E-06 | ^S5,6^ |
| c.116_117dup | p.Ala40Glnfs*7 | LP | P | PVS1 PS3 PM2_supporting PM3_strong PM4 | | | 4.04E-05 | ^S5,7,8^  ^S5,8,9^ |
| c.139G>C | p.Gly47Arg |  | LP | PS1 PM2_supporting PP3 | | | 4.11E-06 | gnomAD |
| c.140G>A | p.Gly47Glu |  | LP | PM2_supporting PM3_supporting PM5 PP3 PP4 | | | 4.12E-06 | ^S10^ |
| c.188G>A | p.Gly63Asp |  | LP | PS3 PM2_supporting PM5 PP3 PP4 | | | 3.98E-06 | ^S1^  ^S1^ |
| c.215A>T | p.Asn72Ile |  | LP | PM2_supporting PM3_strong PM5 PP3 | | | 7.96E-06 | ^S11-13^  ^S12,13^ |
| c.238_239insTTGCCAA | p.Gly80Valfs*90 |  | P | PVS1 PM2_supporting PM4 | | | 3.98E-06 | gnomAD |
| c.245G>A | p.Gly82Glu | LP | P | PS3 PM2_supporting PM3_strong PP3 PP4 | | | 1.99E-05 | ^S14-16^  ^S14-16^ |
| c.302T>C | p.Leu101Pro | P/LP | P | PS3 PS4 PM2_supporting PP1 PP3 | | | 3.2E-05 | ^S3^  ^S3^ |
| c.322T>C | p.Trp108Arg | P/LP | P | PS3 PM2_supporting PM3_strong PP3 PP4 PP5 | | | 4.04E-06 | ^S6,17,18^ |
| c.346G>A | p.Gly116Arg | LP | P | PS3 PS5 PM2_supporting PM3_strong PP3 PP4 | | | 1.88E-05 | ^S19^  ^S19^ |
| c.352C>A | p.Arg118Ser |  | LP | PM2_supporting PM5 PP3 PP4 | | | 4.37E-06 | ^S1^ ^S20^  ^S1^ ^S20^ |
| c.352C>T | p.Arg118Cys | Uncertain | LP | PS3 PM2_supporting PP3 PP4 | | | 6.91E-05 | ^S20^  ^S20^ |
| c.358G>A | p.Gly120Arg | Uncertain | LP | PS3 PM2_supporting PM3_strong PP3 PP5 | | | 4.49E-06 | ^S20,21^  ^S20^ ^S21^ |
| c.359-2A>G | Splice variant |  | LP | PVS1 PM2_supporting |  | | 6.33E-06 | gnomAD |
| c.423+2T>C | Splice variant |  | LP | PVS1 PM2_supporting | |  | 6.26E-06 | gnomAD |
| c.455T>C | p.Phe152Ser |  | VUS | PM2_supporting PM5 PP3 | | | 3.19E-05 | ClinVar |
| c.455T>G | p.Phe152Cys |  | VUS | PM2_supporting PM5 PP3 | | | 3.19E-05 | ClinVar |
| c.469G>C | p.Glu157Gln |  | VUS | PM2_supporting PP3 PP6 | | | 4.38E-06 | ^S19,22^  ^S19^ ^S22^ |
| c.473C>T | p.Ser158Leu | P/LP | LP | PM2_supporting PM3_strong PP3 PP4 PP5 | | | 4.41E-06 | ^S1^  ^S1^ ^S23^ |
| c.481G>T | p.Gly161Cys | P/LP | P | PS3 PM2_supporting PM3_strong PP3 PP4 PP5 | | | 1.55E-05 | ^S24^  ^S24^ |
| c.508G>A | p.Gly170Arg | P/LP | P | PS3 PS4 PM2_supporting PM3_very strong PP1 PP3 | | | 0.000562 | ^S3,25,26^  ^S27^ |
| c.525-2A>T | Splice variant |  | P | PVS1 PM2_supporting PM3 PP3 | | | 4.01E-06 | RKSC |
| c.568G>A | p.Gly190Arg | P/LP | P | PS3 PM2_supporting PM3_strong PP3 PP4 PP5 | | | 4.41E-05 | ^S1,28^  ^S1,28^ |
| c.569_570insA | p.Thr191AspFS*34 |  | LP | PVS1 PM2_supporting | |  | 3.19E-05 | gnomAD |
| c.595+1G>A | Splice variant |  | LP | PVS1 PM2_supporting | | | 4.02E-06 | gnomAD |
| c.597_601del | p.Ile200HisFS*23 |  | LP | PVS1 PM2_supporting | |  | 3.98E-06 | gnomAD |
| c.601G>A | p.Asp201Asn | Uncertain | LP | PM1 PM2_supporting PM5 PP3 | | | 7.97E-06 | ClinVar |
| c.622C>T | p.Gln208* |  | P | PVS1 PM2_supporting PP3 | | | 3.18E-05 | gnomAD |
| c.627G>T | p.Lys209Asn |  | LP | PS3 PM1 PM2_supporting PM3_supporting | | | 3.98E-06 | ^S29^  ^S29^ |
| c.638C>A | p.Ala213Asp |  | VUS | PM2_supporting PM3_supporting PP3 PP4 | | | 3.19E-05 | ^S20^  ^S20^ |
| c.653C>T | p.Ser218Leu | P/LP | LP | PS3 PM1 PM2_supporting PP3 | | | 1.06E-05 | ^S9,16,18,30^  ^S18,30^ |
| c.681-1G>A | Splice variant |  | LP | PVS1 PM2_supporting | |  | 3.98E-06 | gnomAD |
| c.697C>T | p.Arg233Cys | P/LP | P | PS3 PM2_supporting PM3_strong PP3 PP4 PP5 | | | 5.57E-05 | ^S1^  ^S31^ |
| c.698G>A | p.Arg233His | P/LP | P | PS3 PM2_supporting PM3_strong PP3 PP5 | | | 2.12E-05 | ^S8^ ^S16^ ^S5^ |
| c.751T>C | p.Trp251Arg |  | LP | assigned by submitter to ClinVar | |  | 3.98E-06 | ClinVar |
| c.774G>T | p.Arg258Ser |  | VUS | PM2_supporting PP3 PP4 | |  | 2.12E-05 | ^S20^  ^S20^ |
| c.777-1G>C | Splice variant | P/LP | P | PVS1 PS3 PM2_supporting PM3_strong | | | 1.59E-05 | ^S9^  ^S9^ |
| c.781C>G | p.His261Asp | Uncertain | LP | PS1 PM2_supporting PM3 | | | 3.98E-06 | ^S1^  ^S1^ |
| c.822G>C | p.Glu274Asp | Uncertain | P | PS3 PM2_supporting PM3 PP1 PP4 | | | 5.66E-05 | ^S8^ ^S6^ ^S32^ ^S3^ ^S1^  ^S8^ ^S6,32^ ^S1,3^ |
| c.824G>T | p.Ser275Ile |  | LP | PM2_supporting PM5 PP3 PP4 | | | 3.98E-06 | ^S20^  ^S20^ |
| c.858C>G | p.Asn286Lys |  | VUS | PM2_supporting BP4 | |  | 6.55E-06 | ^S19,22^  ^S19^ ^S22^ |
| c.866G>A | p.Arg289His | Conflicting | VUS | PM2_supporting PM3 BP2 BP4 | | | 0.001024 | ^S8^ ^S10^ ^S1^ ^S8^ ^S10^ |
| c.941C>T | p.Pro314Leu |  | VUS | PM2_supporting PP4 | |  | 0.000203 | ^S10^  ^S10^ |
| c.949C>T | p.Arg317Trp | Uncertain | LP | PM2_supporting PM3_strong PP3 PP4 PP5 | | | 3.93E-05 | ^S1^ ^S33^  ^S1^ ^S33^ |
| c.976del | p.Val326Tyrfs*15 | P/LP | P | PVS1 PS3 PM2_supporting PM3_strong | | | 8.01E-06 | ^S34-36^  ^S34,35^ |
| c.1048G>A | p.Gly350Ser |  | VUS | PM2_supporting PM5 PP3 | | | 0.000145 | ClinVar |
| c.1049G>A | p.Gly350Asp | LP | P | PS3 PM2_supporting PM3_strong PP3 PP5 | | | 4.39E-05 | ^S16,17,30^  ^S17^ ^S30^ ^S3^ |
| c.1078C>T | p.Arg360Trp |  | LP | PM2_supporting PM3_supporting PM5 PP3 PP4 | | | 1.2E-05 | ^S1^  ^S1^ |
| c.1079G>A | p.Arg360Gln | LP | P | PS3 PS5 PM2_supporting PP3 PP5 | | | 5.91E-06 | ^S37,38^  ^S3^ ^S8^ |
| c.1094G>C | p.Gly365Ala |  | VUS | PM2_supporting PM5 PP3 PP4 | | | 5.38E-06 | RKSC |

Variants without a reference were curated based on data from ClinVar, RKSC or represent unpublished data from the authors.

ACMG: American College of Medical Genetics; gnomAD: Genome Aggregation Database; LP: likely pathogenic variant; P: pathogenic variant; RKSC: Rare Kidney Stone Consortium; VUS: variant of unknown significance.

**Table S5:** Classification of *GRHPR* variants yielded from gnomAD v.2.1.1 before and after manual review.

| **Variant** | **Protein Consequence** | **Clin Var classification** | **After manual classification** | **ACMG criteria** | **Allele Frequency** | **Reference** |
| --- | --- | --- | --- | --- | --- | --- |
| c.34A>G | p.Thr12Ala | Conflicting | VUS | PM1PM2_supportingPP3 | 6.29112E-05 | gnomAD |
| c.139C>T | p.Arg47Ter | Conflicting | P | PVS1 PM2_supporting PP3 | 3.91122E-05 | ClinVar |
| c.154del | p.Ala52ProfsTer56 | P/LP | P | PVS1 PM2_supporting PP3 | 9.48524E-06 | ClinVar |
| c.286C>T | p.Arg96Cys | Conflicting | VUS | PM2_supporting PM5 PP3 | 0.000685857 | ClinVar |
| c.287G>A | p.Arg96His |  | LP | PM1 PM2_supporting PM5 PP3 | 3.97671E-06 | ^S39^ ^40^ |
| c.287G>T | p.Arg96Leu |  | LP | PM1 PM2_supporting PP3 PP4 PP5 | 3.97671E-06 | OxalEurope |
| c.288-1G>C | Splice variant |  | LP | PVS1PM2_supporting | 3.9764E-06 | gnomAD |
| c.337G>A | p.Glu113Lys | LP | LP | PS3 PM1 PM2_supporting PM3_supporting  PP3 | 3.97646E-06 | ^S1,41^  ^S41^ ^S1^ |
| c.348del | p.Ser117ProfsTer17 |  | LP | PVS1PM2_supporting | 3.97649E-06 | gnomAD |
| c.351_373del | p.Leu118ValfsTer65 |  | LP | PVS1PM2_supporting PM4 | 3.97655E-06 | gnomAD |
| c.370C>T | p.Arg124Cys |  | LP | PS3 PM2_supporting PM3_strong  PP3 | 1.98837E-05 | ^S19,42^  ^S42^ ^S19^ |
| c.404+3_404+6del | Splice variant | P/LP | P | PVS1PS3 PM2_supporting PM3_strong | 2.78529E-05 | ^S43^ ^S44^ |
| c.412T>C | p.Trp138Arg |  | VUS | PM1 PM2_supporting PP3 | 3.98816E-06 | ^S19,22^  ^S19^ ^S22^ |
| c.454dup | p.Thr152AsnfsTer39 | P/LP | P | PVS1 PM2_supporting PP4 | 7.98907E-06 | ^S1,45^  ^S1^ ^S45^ |
| c.501del | p.Ile168LeufsTer5 |  | P | PSV1, PM2_supporting, PM3_supporting, PP4. | 3.97684E-06 | ^S20^  ^S20^ |
| c.536_537del | p.Arg179IlefsTer11 |  | LP | PVS1PM2_supporting | 7.95361E-06 | gnomAD |
| c.598+2del | Splice variant |  | LP | PVS1PM2_supporting | 3.9854E-06 | gnomAD |
| c.598+2dup | Splice variant |  | LP | PVS1PM2_supporting | 7.9708E-06 | gnomAD |
| c.626C>T | p.Ser209Phe |  | LP | PM1 PM2_supporting PM3_strong PP3 PP4 | 1.061E-05 | ^S1,46^  ^S1^ ^S46^ |
| c.735-1G>A | Splice variant | LP | P | PVS1 PM2_supporting PM3_strong PP4 | 1.59137E-05 | ^S21^  ^S21^ |
| c.735-2A>G | Splice variant |  | LP | PVS1PM2_supporting | 3.97836E-06 | gnomAD |
| c.742G>A | p.Val248Ile | Uncertain | VUS | PM2_supporting PM5 | 4.95057E-05 | ClinVar |
| c.766C>T | p.Gln256Ter |  | P | PVS1 PM2_supporting PP3 | 3.97662E-06 | gnomAD |
| c.769dup | p.Ala257GlyfsTer6 |  | P | PVS1 PM2_supporting PM4 | 3.97655E-06 | ^S1^  ^S1^ |
| c.824del | p.Pro275HisfsTer9 |  | P | PVS1 PM2 _supporting PM4 | 3.97655E-06 | gnomAD |
| c.847del | p.Leu283Ter |  | P | PVS1 PM2_supporting PM4 | 1.19304E-05 | gnomAD |
| c.904C>T | p.Arg302Cys | P/LP | P | PS3 PM2_supporting PM3_moderate PP3 PP4 PP5 | 2.47469E-05 | ^S44^  ^S44^ |
| c.866-2A>G | Splice variant | Uncertain | LP | PVS1 PM2_supporting | 6.36641E-05 | gnomAD |
| c.955G>T | p.Gly319Trp | Conflicting | VUS | PM1PM2_supporting PP3 | 0.000176752 | ^S43^ |

Variants without a reference were curated based on data from ClinVar, RSKC or represent unpublished data from the authors.

ACMG: American College of Medical Genetics; gnomAD: Genome Aggregation Database; LP: likely pathogenic variant; P: pathogenic variant; RKSC: Rare Kidney Stone Consortium; VUS: variant of unknown significance.

**Table S6:** Classification of *HOGA1* variants yielded from gnomAD v.2.1.1 before and after manual review.

| **Variant** | **Protein Consequence** | **ClinVar classification** | **After manual classification** | **ACMG criteria** | **Allele Freq** | **References** |
| --- | --- | --- | --- | --- | --- | --- |
| c.10_11insTGGTC | p.Pro4LeufsTer9 | P/LP | P | PVS1 PM2_supporting PM3 | 4.02E-06 | ^S47^ |
| c.41T>A | p.Leu14Gln |  | VUS | PM1 PM2_supporting PP3 | 7.1E-06 | ^S47^ |
| c.103A>G | p.Ile35Val |  | VUS | PM1 PM3_strong PP4 | 2.13E-05 | ^S48^ |
| c.107C>T | p.Ala36Val | Conflicting | LP | PS3 PM2_supporting PM3_strong PP4 | 0.000167 | ^S8,49,50^ |
| c.110G>A | p.Gly37Asp |  | P | PS1 PM1 PM2_supporting PM5 PP3 PP4 | 8E-06 | ^S42,51,52^ |
| c.110G>T | p.Gly37Val | Uncertain | LP | PM1 PM2_supporting PM5 PP3 | 1.2E-05 | ^S8^ |
| c.122dup | p.Val42CysfsTer47 | P/LP | LP | PVS1PM2_supporting | 4.01E-06 | ClinVar |
| c.123del | p.Val42Ter | P/LP | P | PVS1 PM2_supporting PM3_supporting | 3.2E-05 | ClinVar |
| c.134C>G | p.Pro45Arg | Uncertain | LP | PM1 PM2_supporting PM5 PP3 | 2E-05 | ClinVar |
| c.208C>T | p.Arg70Ter | Conflicting | P | PVS1 PM2_supporting PM3_strong | 4.03E-05 | ^S8^ ^S42,52,53^  ^S8,42,52,53^ |
| c.212-1G>A | splice variant |  | LP | PVS1 PM2_supporting | 3.98E-06 | gnomAD |
| c.221T>G | p.Val74Gly | Conflicting | LP | PM2_supporting PM3_moderate PP3 PP4 PP5 | 1.06E-05 | ^S54^  ^S54^ |
| c.234T>A | p.Asn78Lys |  | LP | PM2_supporting PM3_moderate PP3 PP4 | 2.78E-05 | ^S20^  ^S20^ |
| c.238G>T | p.Glu80Ter |  | P | PVS1 PM2_supporting PP3 | 3.98E-06 | gnomAD |
| c.250del | p.Leu84Ter |  | LP | PVS1 PM2_supporting | 3.98E-06 | gnomAD |
| c.251T>C | p.Leu84Pro |  | P | PS3 PM2_supporting PM3_moderate PP3 PP4 | 3.98E-06 | ^S55^  ^S55^ |
| c.266G>A | p.Arg89His |  | LP | PM1 PM2_supporting PM3_strong PP3 PP4 | 1.99E-05 | ^S47^  ^S47^ |
| c.289C>T | p.Arg97Cys | Conflicting | LP | PS3 PM2_supporting PM3_strong PP3 PP4 | 6.01E-05 | ^S47,56-58^  ^S47,56-58^ |
| c.290G>T | p.Arg97Leu | LP | VUS | PM2_supporting PM5 PP3 | 3.98E-06 | ClinVar |
| c.337G>A | p.Glu113Lys | Uncertain | P | PS5 PM1 PM2_supporting PM3_moderate PP3 | 7.96E-06 | ^S7^  ^S7^ |
| c.340+1G>A | splice variant |  | LP | PVS1 PM2_supporting | 3.98E-06 | gnomAD |
| c.376G>A | p.Ala126Thr |  | VUS | PM2_supporting PM3_moderate PP3 PP4 | 7.09E-06 | ^S57,59^  ^S47,57^ |
| c.385G>A | p.Gly129Arg | Uncertain | LP | PM2_supporting PM3_strong PP3 PP4 | 1.6E-05 | ^S20^  ^S20^ |
| c.425G>A | p.Arg142His |  | LP | PM2_supporting PM3_moderate PP3 PP4 | 3.19E-05 | OxalEurope |
| c.448del | p.Leu150SerfsTer46 | P/LP | LP | PVS1PM2_supporting | 1.06E-05 | ClinVar |
| c.469-1G>C | splice variant |  | LP | PVS1PM2_supporting | 3.98E-06 | gnomAD |
| c.533T>A | p.Leu178Gln | Uncertain | LP | PM2_supporting PM3 PP3 PP4 | 3.98E-06 | ^S20^  ^S20^ |
| c.580G>A | p.Gly194Ser |  | LP | PM1 PM2_supporting PM3_moderate PP3 PP4 | 3.98E-06 | ^S51,57^  ^S51,57^ |
| c.634A>G | p.Thr212Ala | Uncertain | LP | PM1 PM2_supporting PM3_strong PM5 PP3 PP4 | 1.19E-05 | ^S60^  ^S60^ |
| c.651dup | p.Gln218SerfsTer57 |  | LP | PVS1 PM2_supporting | 3.98E-06 | gnomAD |
| c.700+2T>A | splice variant |  | LP | PVS1 PM2_supporting | 3.98E-06 | gnomAD |
| c.713del | p.Gly238AlafsTer42 | Uncertain | LP | PVS1 PM2_supporting | 3.99E-06 | RKSC |
| c.720C>A | p.Cys240Ter |  | LP | PVS1 PM2_supporting | 7.98E-06 | gnomAD |
| c.733G>T | p.Val245Phe | Uncertain | LP | PM1 PM2_supporting PM3_strong PM5 PP3 | 1.77E-05 | ^S20^  ^S20^ |
| c.743del | p.Ala248ValfsTer32 |  | LP | PVS1 PM2_supporting | 3.99E-06 | gnomAD |
| c.754C>T | p.Gln252Ter |  | P | PVS1 PM2_supporting PP3 | 4E-06 | gnomAD |
| c.763C>T | p.Arg255Ter | P/LP | P | PVS1 PM2_supporting PM3_moderate PP3 PP4 | 1.78E-05 | ^S61^  ^S61^ |
| c.769_770del | p.Cys257LeufsTer17 |  | P | PVS1 PM2_supporting PM3 | 1.2E-05 | OxalEurope |
| c.769T>G | p.Cys257Gly | P/LP | LP | PM1 PM2_supporting PM3_strong PP1 PP4 | 6.76E-05 | ^S42,53^  ^S42,53^ |
| c.776C>T | p.Thr259Met | Conflicting | VUS | PM1 PP3 | 8.20E-05 | gnomAD |
| c.785G>A | p.Trp262Ter |  | P | PVS1 PM2_supporting PP3 | 8.04E-06 | gnomAD |
| c.787G>T | p.Glu263Ter |  | P | PVS1 PM2_supporting PP3 | 2.01E-05 | gnomAD |
| c.796C>T | p.Gln266Ter | LP | P | PVS1 PM2_supporting PP3 PP5 | 4.04E-06 | ClinVar |
| c.811C>T | p.Arg271Cys | Conflicting | VUS | PM2_supporting PP3 PP4 | 0.000101 | ^S52,62^  ^S52,62^ |
| c.812G>A | p.Arg271His | P/LP | LP | PM1 PM2_supporting PM3_supporting PP3 PP4 | 2.03E-05 | ^S52^  ^S52^ |
| c.818T>C | p.Ile273Thr | Uncertain | LP | PM1 PM2_supporting PM3_moderate PP3 PP4 | 2.44E-05 | ^S20^  ^S20^ |
| c.833C>A | p.Ala278Glu |  | VUS | PM1 PM2_supporting PM3_supporting PP3 | 8.27E-06 | ^S59^  ^S47^ |
| c.834+1G>T | splice variant | LP | P | PVS1 PM2_supporting PM3_strong PP4 | 6.62E-05 | ^S51,53^ ^S48,62,63^ |
| c.845G>A | p.Arg282His |  | VUS | PM1 PM2_supporting PP3 | 8.00E-06 | ^S48^  ^S48^ |
| c.860G>A | p.Gly287Glu |  | P | PS5 PM1 PM3_moderate PM5 PP3 | 2E-05 | ^S55^  ^S55^ |
| c.860G>T | p.Gly287Val | P/LP | P | PS3 PM1 PM3_strong PP3 PP4 | 0.000108 | ^S49,56,58^ |
| c.881G>A | p.Trp294Ter |  | P | PVS1 PM2_supporting PP3 | 3.99E-06 | ^S47^ |
| c.908G>A | p.Arg303His |  | LP | PM1 PM3_supporting PM5 PP3 | 4.96E-05 | ^S59^  ^S47^ |
| c.926T>C | p.Leu309Pro |  | LP | PM1 PM2_supporting PM3_supporting PP3 PP4 | 3.98E-06 | RKSC |
| c.940G>T | p.Glu314Ter |  | P | PVS1 PM2_supporting PP3 | 3.98E-06 | gnomAD |
| c.943_954dup | p.Glu315_Arg318dup | Uncertain | LP | PM2_supporting PM3_supporting PM4 PP3 PP4 | 3.18E-05 | ^S20,47^  ^S20,47^ |
| c.949_956dup | p.Met319IlefsTer22 |  | LP | PVS1 PM2_supporting | 3.98E-06 | gnomAD |
| c.953G>A | p.Arg318His | Uncertain | VUS | PM2_supporting PM3_supporting PP3 PP4 | 1.42E-05 | ^S8^  ^S8^ |
| c.977G>A | p.Trp326Ter |  | LP | PVS1 PM2_supporting PP3 | 4.02E-06 | gnomAD |

Variants without a reference were curated based on data from ClinVar, RSKC or represent unpublished data from the authors.

ACMG: American College of Medical Genetics; gnomAD: Genome Aggregation Database; LP: likely pathogenic variant; P: pathogenic variant; RKSC: Rare Kidney Stone Consortium; VUS: variant of unknown significance.

**Figure S1:** Global and ethnic-specific PH carrier frequencies with variants of unknown significance (VUS) following comprehensive curation and reclassification. (A) PH1 carrier frequency when including pathogenic variants, likely pathogenic variants, and VUS. (B) PH2 carrier frequency when including pathogenic variants, likely pathogenic variants, and VUS. (C) PH3 carrier frequency when including pathogenic variants, likely pathogenic variants, and VUS. Deeper heatmap colors indicate greater prevalence.

(A)


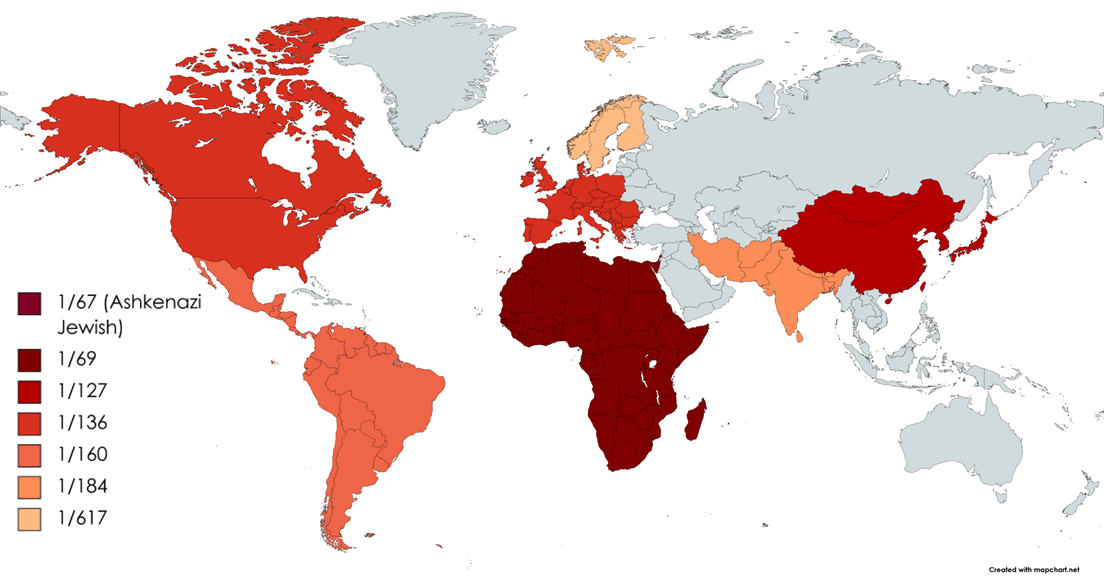


(B)


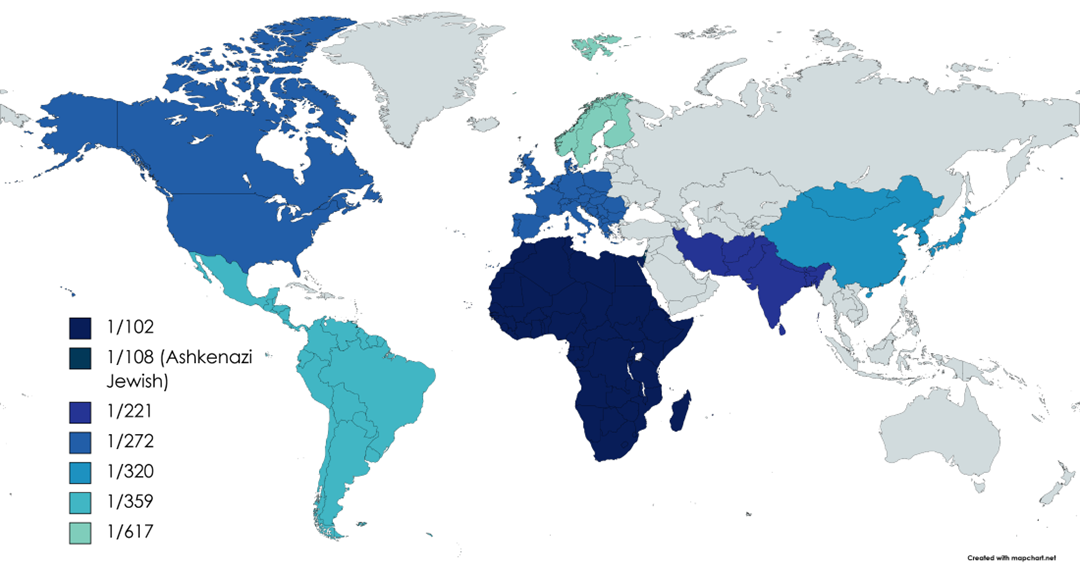


(C)


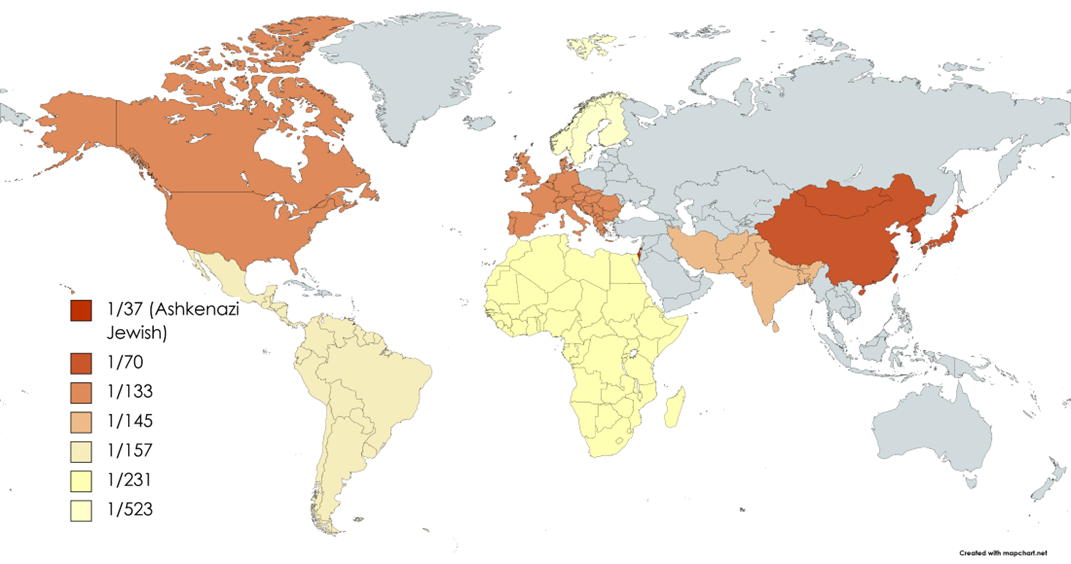


## **SUPPLEMENTARY REFERENCES**

S1. Hopp K, Cogal AG, Bergstralh EJ, et al. Phenotype-genotype correlations and estimated carrier frequencies of primary hyperoxaluria. *J Am Soc Nephrol* 2015;**26**:2559–70. doi:10.1681/ASN.2014070698

S2. Zhao F, Li J, Tang L, Li C, Wang W, Ning C. Characteristics of the genotype and phenotype in Chinese primary hyperoxaluria type 1 populations. *Urolithiasis* 2021;**49**:17–25. doi:10.1007/s00240-020-01201-x

S3. Williams EL, Acquaviva C, Amoroso A, et al. Primary hyperoxaluria type 1: update and additional mutation analysis of the *AGXT* gene. *Hum Mutat* 2009;**30**:910–7. doi:10.1002/humu.21021

S4. Dindo M, Mandrile G, Conter C, et al. The ILE56 mutation on different genetic backgrounds of alanine:glyoxylate aminotransferase: Clinical features and biochemical characterization. *Mol Genet Metab* 2020;**131**:171–80. doi:10.1016/j.ymgme.2020.07.012

S5. Williams E, Rumsby G. Selected exonic sequencing of the AGXT gene provides a genetic diagnosis in 50% of patients with primary hyperoxaluria type 1. *Clin Chem* 2007;**53**:1216–21. doi:10.1373/clinchem.2006.084434

S6. Lage MD, Pittman AM, Roncador A, Cellini B, Tucker CL. Allele-specific characterization of alanine: glyoxylate aminotransferase variants associated with primary hyperoxaluria. *PLoS One* 2014;**9**:e94338. doi:10.1371/journal.pone.0094338

S7. Clifford-Mobley O, Hewitt L, Rumsby G. Simultaneous analysis of urinary metabolites for preliminary identification of primary hyperoxaluria. *Ann Clin Biochem* 2016;**53**:485–94. doi:10.1177/0004563215606158

S8. Williams EL, Bagg EA, Mueller M, Vandrovcova J, Aitman TJ, Rumsby G. Performance evaluation of Sanger sequencing for the diagnosis of primary hyperoxaluria and comparison with targeted next generation sequencing. *Mol Genet Genomic Med* 2015;**3**:69–78. doi:10.1002/mgg3.118

S9. Coulter-Mackie MB, Lian Q, Applegarth D, Toone J. The major allele of the alanine:glyoxylate aminotransferase gene: nine novel mutations and polymorphisms associated with primary hyperoxaluria type 1. *Mol Genet Metab* 2005;**86**:172–8. doi:10.1016/j.ymgme.2005.05.005

S10. Murad H, Alhalabi MB, Dabboul A, et al. Molecular analysis of the AGXT gene in Syrian patients suspected with primary hyperoxaluria type 1. *BMC Med Genomics* 2021;**14**:146. doi:10.1186/s12920-021-00996-x

S11. Wang C, Lu J, Lang Y, et al. Two novel AGXT mutations identified in primary hyperoxaluria type-1 and distinct morphological and structural difference in kidney stones. *Sci Rep* 2016;**6**:33652. doi:10.1038/srep33652

S12. Lin JA, Liao X, Wu W, Xiao L, Liu L, Qiu J. Clinical analysis of 13 children with primary hyperoxaluria type 1. *Urolithiasis* 2021;**49**:425–31. doi:10.1007/s00240-021-01249-3

S13. Cai R, Lin M, Chen Z, et al. Primary hyperoxaluria diagnosed after kidney transplantation failure: lesson from 3 case reports and literature review. *BMC Nephrol* 2019;**20**:224. doi:10.1186/s12882-019-1402-2

S14. Purdue PE, Lumb MJ, Allsop J, Minatogawa Y, Danpure CJ. A glycine-to-glutamate substitution abolishes alanine:glyoxylate aminotransferase catalytic activity in a subset of patients with primary hyperoxaluria type 1. *Genomics* 1992;**13**:215–8. doi:10.1016/0888-7543(92)90225-h

S15. Cellini B, Bertoldi M, Montioli R, Paiardini A, Borri Voltattorni C. Human wild-type alanine:glyoxylate aminotransferase and its naturally occurring G82E variant: functional properties and physiological implications. *Biochem J* 2007;**408**:39–50. doi:10.1042/BJ20070637

S16. Coulter-Mackie MB, Lian Q, Applegarth DA, Toone J, Waters PJ, Vallance H. Mutation-based diagnostic testing for primary hyperoxaluria type 1: survey of results. *Clin Biochem* 2008;**41**:598–602. doi:10.1016/j.clinbiochem.2008.01.018

S17. von Schnakenburg C, Rumsby G. Identification of new mutations in primary hyperoxaluria type 1 (PH1). *J Nephrol* 1998;**11**(Suppl 1):15–7.

S18. Coulter-Mackie MB, Lian Q. Consequences of missense mutations for dimerization and turnover of alanine:glyoxylate aminotransferase: study of a spectrum of mutations. *Mol Genet Metab* 2006;**89**:349–59. doi:10.1016/j.ymgme.2006.07.013

S19. Hashmi S, Abid A, Sultan S, Shekhani SS, Lanewala AA, Zafar MN. Primary hyperoxaluria and genetic linkages: an insight into the disease burden from Pakistan. *Urolithiasis* 2022;**50**:439–45. doi:10.1007/s00240-022-01338-x

S20. Cogal AG, Arroyo J, Shah RJ, et al. Comprehensive genetic analysis reveals complexity of monogenic urinary stone disease. *Kidney Int Rep* 2021;**6**:2862–84. doi:10.1016/j.ekir.2021.08.033

S21. Pinapala A, Garg M, Kamath N, Iyengar A. Clinical and genetic profile of Indian children with primary hyperoxaluria. *Indian J Nephrol* 2017;**27**:222–4. doi:10.4103/0971-4065.202831

S22. Abid A, Raza A, Khan AR, et al. Primary hyperoxaluria: Comprehensive mutation screening of the disease causing genes and spectrum of disease-associated pathogenic variants. *Clin Genet* 2023;**103**:53–66. doi:10.1111/cge.14240

S23. Cai Z, Ding M, Chen R, Zhu J, Li L, Wu X. Primary hyperoxaluria diagnosed after kidney transplantation: a case report and literature review. *BMC Nephrol* 2021;**22**:393. doi:10.1186/s12882-021-02546-0

S24. Oppici E, Roncador A, Montioli R, Bianconi S, Cellini B. Gly161 mutations associated with primary hyperoxaluria type I induce the cytosolic aggregation and the intracellular degradation of the apo-form of alanine:glyoxylate aminotransferase. *Biochim Biophys Acta* 2013;**1832**:2277–88. doi:10.1016/j.bbadis.2013.09.002

S25. Danpure CJ. The molecular basis of alanine: glyoxylate aminotransferase mistargeting: the most common single cause of primary hyperoxaluria type 1. *J Nephrol* 1998;**11**(Suppl 1):8–12.

S26. Mandrile G, van Woerden CS, Berchialla P, et al. Data from a large European study indicate that the outcome of primary hyperoxaluria type 1 correlates with the AGXT mutation type. *Kidney Int* 2014;**86**:1197–1204. doi:10.1038/ki.2014.222

S27. Danpure CJ, Purdue PE, Fryer P, et al. Enzymological and mutational analysis of a complex primary hyperoxaluria type 1 phenotype involving alanine:glyoxylate aminotransferase peroxisome-to-mitochondrion mistargeting and intraperoxisomal aggregation. *Am J Hum Genet* 1993;**53**:417–32.

S28. M'Dimegh S, Omezzine A, Hamida-Rebai MB, et al. Identification of a novel AGXT gene mutation in primary hyperoxaluria after kidney transplantation failure. *Transpl Immunol* 2016;**39**:60–5. doi:10.1016/j.trim.2016.08.008

S29. Du DF, Li QQ, Chen C, et al. Updated genetic testing of primary hyperoxaluria type 1 in a Chinese population: Results from a single center study and a systematic review. *Curr Med Sci* 2018;**38**:749–57. doi:10.1007/s11596-018-1941-y

S30. Oppici E, Montioli R, Lorenzetto A, Bianconi S, Borri Voltattorni C, Cellini B. Biochemical analyses are instrumental in identifying the impact of mutations on holo and/or apo-forms and on the region(s) of alanine:glyoxylate aminotransferase variants associated with primary hyperoxaluria type I. *Mol Genet Metab* 2012;**105**:132–40. doi:10.1016/j.ymgme.2011.09.033

S31. von Schnakenburg C, Rumsby G. Primary hyperoxaluria type 1: a cluster of new mutations in exon 7 of the AGXT gene. *J Med Genet* 1997;**34**:489–92. doi:10.1136/jmg.34.6.489

S32. Pittman AM, Lage MD, Poltoratsky V, et al. Rapid profiling of disease alleles using a tunable reporter of protein misfolding. *Genetics* 2012;**192**:831–42. doi:10.1534/genetics.112.143750

S33. Dieudonne Y, Eprinchard L, Leon E, et al. Paraplegia as a presentation of primary hyperoxaluria. *CEN Case Rep* 2018;**7**:313–5. doi:10.1007/s13730-018-0349-7

S34. Pirulli D, Puzzer D, Ferri L, et al. Molecular analysis of hyperoxaluria type 1 in Italian patients reveals eight new mutations in the alanine: glyoxylate aminotransferase gene. *Hum Genet* 1999;**104**:523–5. doi:10.1007/s004390050998

S35. Amoroso A, Pirulli D, Florian F, et al. AGXT gene mutations and their influence on clinical heterogeneity of type 1 primary hyperoxaluria. *J Am Soc Nephrol* 2001;**12**:2072–9. doi:10.1681/ASN.V12102072

S36. van der Hoeven SM, van Woerden CS, Groothoff JW. Primary hyperoxaluria type 1, a too often missed diagnosis and potentially treatable cause of end-stage renal disease in adults: results of the Dutch cohort. *Nephrol Dial Transplant* 2012;27:3855–62. doi:10.1093/ndt/gfs320

S37. M'Dimegh S, Omezzine A, M'Barek I, et al. Mutational analysis of Agxt in Tunisian population with primary hyperoxaluria type 1. *Ann Hum Genet* 2017;**81**:1–10. doi:10.1111/ahg.12178

S38. Daga A, Majmundar AJ, Braun DA, et al. Whole exome sequencing frequently detects a monogenic cause in early onset nephrolithiasis and nephrocalcinosis. *Kidney Int* 2018;**93**:204–13. doi:10.1016/j.kint.2017.06.025

S39. Valoti E, Alberti M, Carrara C, et al. Hemolytic uremic syndrome in an infant with primary hyperoxaluria type II: an unreported clinical association. *Nephron* 2019;**142**:264–70. doi:10.1159/000497823

S40. Birtel J, Herrmann P, Garrelfs SF, et al. The ocular phenotype in primary hyperoxaluria type 1. *Am J Ophthalmol* 2019;**206**:184–91. doi:10.1016/j.ajo.2019.04.036

S41. Takayama T, Nagata M, Ozono S, Nonomura K, Cramer SD. A novel mutation in the GRHPR gene in a Japanese patient with primary hyperoxaluria type 2. *Nephrol Dial Transplant* 2007;**22**:2371–74. doi:10.1093/ndt/gfm271

S42. He L, Xu G, Fang X, et al. Identification of 8 novel gene variants in primary hyperoxaluria in 21 Chinese children with urinary stones. *World J Urol* 2019;**37**:1713–21. doi:10.1007/s00345-018-2563-5

S43. Webster KE, Ferree PM, Holmes RP, Cramer SD. Identification of missense, nonsense, and deletion mutations in the GRHPR gene in patients with primary hyperoxaluria type II (PH2). *Hum Genet* 2000;**107**:176–85. doi:10.1007/s004390000351

S44. Cregeen DP, Williams EL, Hulton S, Rumsby G. Molecular analysis of the glyoxylate reductase (GRHPR) gene and description of mutations underlying primary hyperoxaluria type 2. *Hum Mutat* 2003;**22**:497. doi:10.1002/humu.9200

S45. Konkolova J, Chandoga J, Kovacik J, et al. Severe child form of primary hyperoxaluria type 2 - a case report revealing consequence of GRHPR deficiency on metabolism. *BMC Med Genet* 2017;**18**:59. doi:10.1186/s12881-017-0421-8

S46. Rios JFN, Zuluaga M, Higuita LMS, et al. Primary hiperoxaluria diagnosed after kidney transplantation: report of 2 cases and literature review. *J Bras Nefrol* 2017;**39**:462–6. doi:10.5935/0101-2800.20170081

S47. Martin-Higueras C, Garrelfs SF, Groothoff JW, et al. A report from the European Hyperoxaluria Consortium (OxalEurope) Registry on a large cohort of patients with primary hyperoxaluria type 3. *Kidney Int* 2021;**100**:621–35. doi:10.1016/j.kint.2021.03.031

S48. Huang L, Qi C, Zhu G, et al. Genetic testing enables a precision medicine approach for nephrolithiasis and nephrocalcinosis in pediatrics: a single-center cohort. *Mol Genet Genomics* 2022;**297**:1049–61. doi:10.1007/s00438-022-01897-z

S49. Williams EL, Bockenhauer D, van't Hoff WG, et al. The enzyme 4-hydroxy-2-oxoglutarate aldolase is deficient in primary hyperoxaluria type 3. *Nephrol Dial Transplant* 2012;**27**:3191–5. doi:10.1093/ndt/gfs039

S50. Pitt JJ, Willis F, Tzanakos N, Belostotsky R, Frishberg Y. 4-hydroxyglutamate is a biomarker for primary hyperoxaluria type 3. *JIMD Rep* 2015;**15**:1–6. doi:10.1007/8904_2013_291

S51. Ventzke A, Feldkotter M, Wei A, Becker J, Beck BB, Hoppe B. Systematic assessment of urinary hydroxy-oxo-glutarate for diagnosis and follow-up of primary hyperoxaluria type III. *Pediatr Nephrol* 2017;**32**:2263–71. doi:10.1007/s00467-017-3731-3

S52. Fang X, He L, Xu G, Lin H, Xu M, Geng H. Nine novel HOGA1 gene mutations identified in primary hyperoxaluria type 3 and distinct clinical and biochemical characteristics in Chinese children. *Pediatr Nephrol* 2019;**34**:1785–90. doi:10.1007/s00467-019-04279-7

S53. Allard L, Cochat P, Leclerc AL, et al. Renal function can be impaired in children with primary hyperoxaluria type 3. *Pediatr Nephrol* 2015;**30**:1807–13. doi:10.1007/s00467-015-3090-x

S54. Beck BB, Baasner A, Buescher A, et al. Novel findings in patients with primary hyperoxaluria type III and implications for advanced molecular testing strategies. *Eur J Hum Genet* 2013;**21**:162–72. doi:10.1038/ejhg.2012.139

S55. Greed L, Willis F, Johnstone L, et al. Metabolite diagnosis of primary hyperoxaluria type 3. *Pediatr Nephrol* 2018;**33**:1443–6. doi:10.1007/s00467-018-3967-6

S56. Belostotsky R, Seboun E, Idelson GH, et al. Mutations in DHDPSL are responsible for primary hyperoxaluria type III. *Am J Hum Genet* 2010;**87**:392–9. doi:10.1016/j.ajhg.2010.07.023

S57. Sikora P, Zaniew M, Grenda R, et al. Still diagnosed too late and under-recognized? The first comprehensive report on primary hyperoxaluria in Poland. *Pol Arch Intern Med* 2020;**130**:1053–63. doi:10.20452/pamw.15698

S58. Riedel TJ, Knight J, Murray MS, Milliner DS, Holmes RP, Lowther WT. 4-Hydroxy-2-oxoglutarate aldolase inactivity in primary hyperoxaluria type 3 and glyoxylate reductase inhibition. *Biochim Biophys Acta* 2012;**1822**:1544–52. doi:10.1016/j.bbadis.2012.06.014

S59. Cardinal HN, Fenster A. Theoretical optimization of a split septaless xenon ionization detector for dual-energy chest radiography. *Med Phys* 1988;**15**:167–80. doi:10.1118/1.596248

S60. Richard E, Blouin JM, Harambat J, et al. Late diagnosis of primary hyperoxaluria type III. *Ann Clin Biochem* 2017;**54**:406–11. doi:10.1177/0004563216677101

S61. Monico CG, Rossetti S, Belostotsky R, et al. Primary hyperoxaluria type III gene HOGA1 (formerly DHDPSL) as a possible risk factor for idiopathic calcium oxalate urolithiasis. *Clin J Am Soc Nephrol* 2011;**6**:2289–95. doi:10.2215/CJN.02760311

S62. Wang W, Liu Y, Kang L, et al. Mutation hot spot region in the HOGA1 gene associated with primary hyperoxaluria type 3 in the Chinese population. *Kidney Blood Press Res* 2019;**44**:743–53. doi:10.1159/000501458

S63. Du Y, Roger VB, Mena J, Kang M, Stoller ML, Ho SP. Structural and chemical heterogeneities of primary hyperoxaluria kidney stones from pediatric patients. *J Pediatr Urol* 2021;**17**:214 e211–214 e211. doi:10.1016/j.jpurol.2020.11.023
